# Transport signatures of an Andreev molecule in a quantum dot–superconductor–quantum dot setup

**DOI:** 10.3762/bjnano.10.36

**Published:** 2019-02-06

**Authors:** Zoltán Scherübl, András Pályi, Szabolcs Csonka

**Affiliations:** 1 Department of Physics and MTA-BME Momentum Nanoelectronics Research Group, Budapest University of Technology and Economics, Budafoki út 8., 1111 Budapest, Hungary; 2 Department of Theoretical Physics and MTA-BME Exotic Quantum Phases ”Momentum” Research Group, Budapest University of Technology and Economics, 1111 Budapest, Hungary

**Keywords:** Andreev bound state, crossed Andreev reflection, elastic co-tunneling, quantum dot

## Abstract

Hybrid devices combining quantum dots with superconductors are important building blocks of conventional and topological quantum-information experiments. A requirement for the success of such experiments is to understand the various tunneling-induced non-local interaction mechanisms that are present in the devices, namely crossed Andreev reflection, elastic co-tunneling, and direct interdot tunneling. Here, we provide a theoretical study of a simple device that consists of two quantum dots and a superconductor tunnel-coupled to the dots, often called a Cooper-pair splitter. We study the three special cases where one of the three non-local mechanisms dominates, and calculate measurable ground-state properties, as well as the zero-bias and finite-bias differential conductance characterizing electron transport through this device. We describe how each non-local mechanism controls the measurable quantities, and thereby find experimental fingerprints that allow one to identify and quantify the dominant non-local mechanism using experimental data. Finally, we study the triplet blockade effect and the associated negative differential conductance in the Cooper-pair splitter, and show that they can arise regardless of the nature of the dominant non-local coupling mechanism. Our results should facilitate the characterization of hybrid devices, and their optimization for various quantum-information-related experiments and applications.

## Introduction

Superconducting hybrid nanodevices provide a promising platform for quantum architectures. While superconductors (SCs) allow for a spatially extended coherent state, nanodevices provide the confinement of electrons into 1D or 0D. The interplay of these properties is a key ingredient of novel promising qubit realizations, such as Majorana qubits [1–2] and Andreev qubits [3].

The basic physical mechanism behind these applications is the Andreev reflection, when a Cooper pair from the SC is transformed to two electrons in the normal conductor. The conversion of Cooper pairs has a special form called crossed Andreev reflection (CAR), when the two electrons originating from the Cooper pair ends up in spatially separated normal parts [4–7].

CAR is also a potential resource for quantum hardware. On one hand, it naturally generates entangled spatially separated electron pairs [7]. On the other hand, several novel topological superconducting proposals are based on CAR processes, such as the poor man’s Majorana setup [8], the Majorana chain [9], Tritops [10–14], Majorana states in graphene [15–17] and devices with even more exotic non-Abelian excitations, such as parafermions [18–20]. CAR was studied experimentally in metallic nanostructures [21–24] and later in so-called Cooper-pair splitter devices, where two quantum dots (QDs) are weakly tunnel-coupled to a superconductor in a QD–SC–QD geometry [25–32]. The QD–SC–QD setup serves also as the basic building block of the poor man’s Majorana setup [8] and the Majorana chain [9].

Strong tunnel coupling between a QD and a SC leads to the formation of Andreev bound states (ABSs) [33–51] via local Andreev reflection (LAR). Due to the charging energy on the QDs, the QD–SC–QD geometry prefers CAR over the LAR and leads to the expectation that CAR hybridizes the states of the two QDs, generating the so-called Andreev molecular state [52–54]. The first experimental realization towards such a state is already reported [55]. However, CAR is not the only coupling mechanism between the QDs. Electrons can be transmitted from one of the QDs to the other via virtual intermediate SC quasiparticle states, via the so-called elastic co-tunneling (EC) process [21,23,56–68]. Furthermore, if there is direct tunnel coupling between the dots, as in certain experimental realizations [69–70], then this interdot coupling (IT) also influences the spectrum and the dynamics.

Several previous works on similar systems completely neglect the possibility of EC processes [7,52–54,71–72]. In other works EC coupling is taken into account [44,61,64–65,67–68,73], but in most of the cases it is defined as a constant coupling term that is equivalent to the IT coupling used here, therefore they can be incorporated to the same term. However, as we demonstrate below, in certain models the EC term is not constant, but it can depend on the on-site energies of the QDs. To keep the generality, we treat the energy-dependent EC and the independent IT term separately.

In an experimental realization of a QD–SC–QD setup, any of the three non-local coupling mechanisms (CAR, EC or IT) could dominate. The focus of this paper is to calculate measurable quantities and explore differences between the individual fingerprints of the three non-local processes, which allow one to identify and quantify the dominant non-local term. In particular, we describe the ground-state properties (phase diagram, average electron occupation) of the system, the zero-bias conductance describing electron transport through the device in the presence of tunnel-coupled normal leads, and the excitation spectrum that is accessible via finite-bias transport measurements.

The paper is organized as follows: First, we introduce the microscopic model of the Cooper-pair splitter, and outline how to derive a simple effective Hamiltonian, which describes the non-local coupling mechanisms as individual Hamiltonian terms, from the microscopic model. Then, we analyze the ground-state properties, and the phase diagram and average electron occupation of the QD–SC–QD system. Subsequently, we outline the transport model describing the setup where each QD is coupled to a normal lead. Finally, we analyze the transport signatures of the different non-local coupling terms via the zero bias conductance, the finite-bias conductance, and the triplet blockade effect.

## Model

### Microscopic Hamiltonian of the proximitized double quantum dot

**Figure 1:**
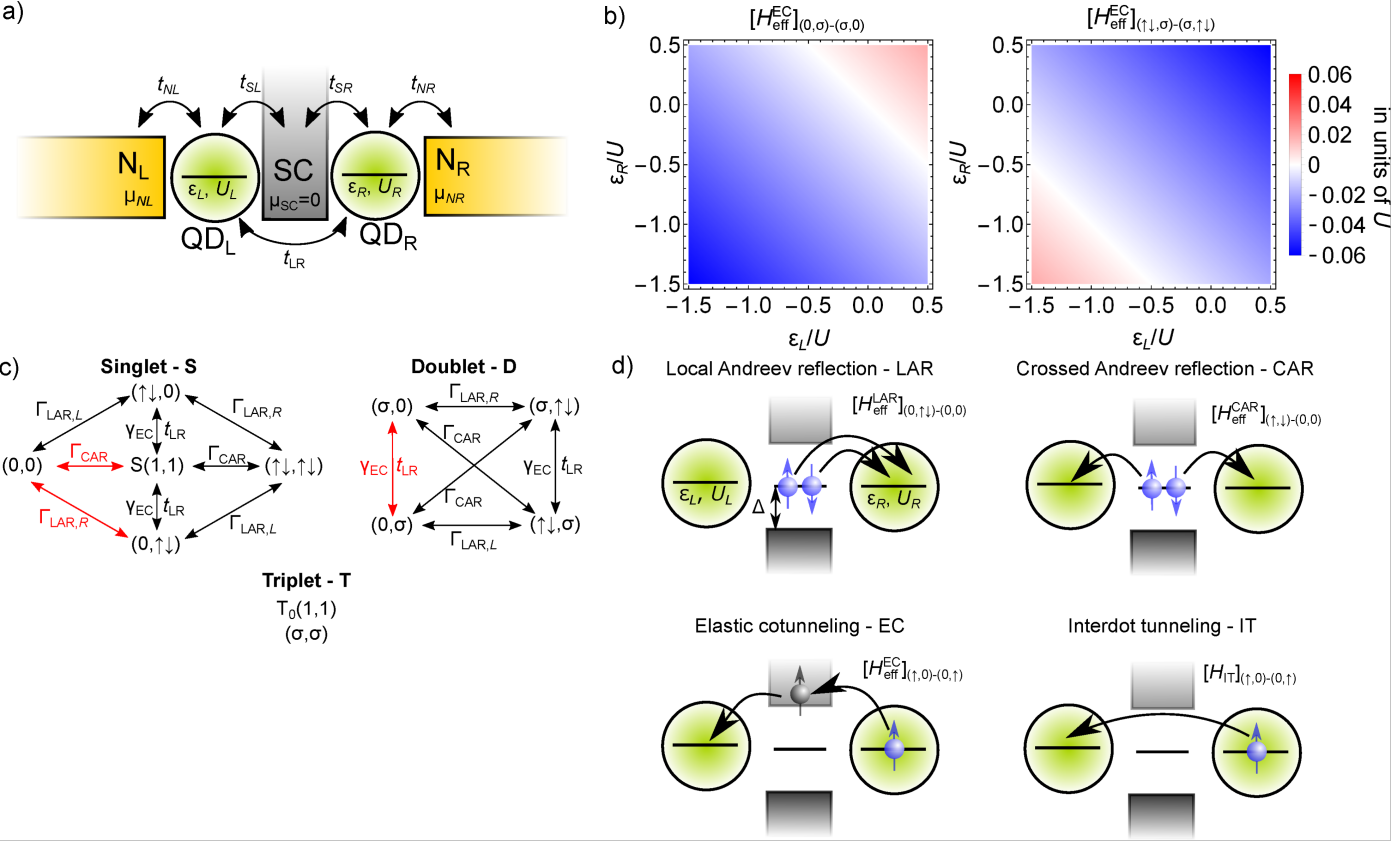
Coherent hybridization between a superconductor and two quantum dots in a Cooper-pair splitter. a) Schematics of the Cooper-pair splitter setup. b) Two examples of the dependence of the EC matrix elements on the on-site energies of the QDs. c) Invariant subspaces of the effective Hamiltonian *H*_eff_ describing the QD–SC–QD setup, also showing the couplings between the basis states. d) Examples of the coupling processes encoded in the effective Hamiltonian *H*_eff_. LAR couples, e.g., state 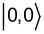 to state 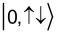 by transferring a Cooper pair from SC to QD_R_. CAR splits a Cooper pair by filling both QDs with one electron with opposite spins. EC transfers an electron from one QD to the other via a virtual intermediate quasiparticle state in SC. IT transfers an electron from one QD to the other without any interaction with SC.

Throughout this work, we study a standard Cooper-pair splitter device. The setup is shown in Figure 1a. It consists of two QDs, each of them tunnel-coupled to its own normal (N) lead, and a common superconducting lead (SC). The Hamiltonian of the system is:

[1]
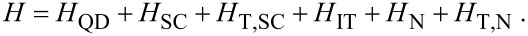


We assume that the level spacings of the dots are large, i.e., each QD has a single spinful orbital, which can be occupied by 0, 1 or 2 electrons. The first term in the Hamiltonian reads as

[2]
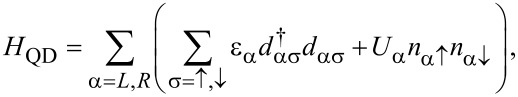


where ε_α_ is the on-site energy of QD_α_ (α = *L*,*R*), *d*_ασ_ (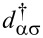) annihilates (creates) an electron on QD_α_ with spin σ, and *U*_α_ is the on-site Coulomb repulsion energy. Note that the interdot Coulomb repulsion in neglected here, since the SC lead between the QDs screens this interaction. Throughout this work, we assume identical Coulomb repulsion energies in the two dots, and use this energy scale *U* = *U_L_* = *U_R_* as the unit of energy.

The SC lead is described by the standard mean-field Bardeen–Cooper–Schrieffer (BCS) Hamiltonian

[3]
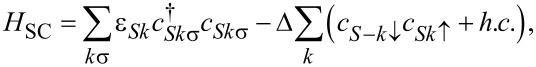


where ε*_Sk_* is the dispersion of conduction electrons in the SC, *c_Skσ_* (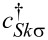) annihilates (creates) an electron in the SC with momentum *k* and spin σ, and Δ is the superconducting order parameter. Only one SC lead is present in our setup, hence Δ is chosen to be real. *H*_SC_ can be diagonalized by a Bogoliubov-transformation, 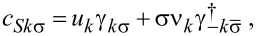 where

[4]
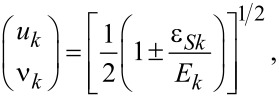


resulting in

[5]
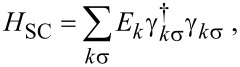


where 
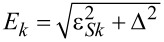
 is the quasi-particle energy.

The Hamiltonian of the normal leads is

[6]
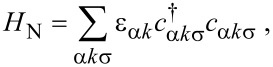


where α = *L*,*R* is the lead index, ε_α_*_k_* is the dispersion relation, and *c*_α_*_k_*_σ_ (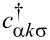) annihilates (creates) an electron with momentum *k* and spin σ in lead N_α_.

Tunneling between the three leads and the two dots is described by the following terms:

[7]
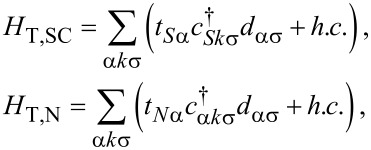


where *H*_T,SC_ describes the tunneling between the QDs and the SC lead, while *H*_T,N_ between the dots and the N leads, with *t_Sα_* (*t_Nα_*) being the tunneling amplitude between the SC (N_α_) lead and QD_α_. Tunneling to the SC will be treated coherently, while tunneling to the N leads is assumed to be weak, and are treated by Fermi’s Golden Rule in the transport model outlined below.

Finally,

[8]
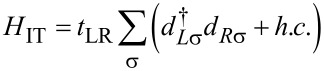


describes interdot tunneling (IT), i.e., direct tunneling between the QDs, with an amplitude *t*_LR_.

### Effective Hamiltonian of the proximitized double quantum dot

The complete Hamiltonian *H* specified above is infinite-dimensional. However, if the temperature *T* is low and the superconducting gap Δ is large, then one may simplify the Hamiltonian by eliminating the superconducting quasiparticles from the description. Technically, this is done by integrating out the quasiparticles using second-order perturbation theory in the SC-QD tunneling term *H*_T,SC_. This procedure yields a 16-dimensional low-energy effective Hamiltonian for the double QD, which describes the superconducting proximity effect of the SC lead on the double QD. Here, we describe this effective Hamiltonian and the procedure to obtain it.

For this, we consider the Hamiltonian without the N leads, *H*_QD_ + *H*_SC_ + *H*_T,SC_ + *H*_IT_. (We will take into account the N leads later to describe transport.) Assuming Δ 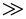 *U* and further neglecting the QD-SC tunneling *H*_T,SC_, the 16-dimensional quasiparticle-free low-energy subspace is energetically well-separated from other states containing a finite number of quasiparticles. The low-energy subspace is spanned by the product basis, the products of particle-number eigenstates of each QD, namely,


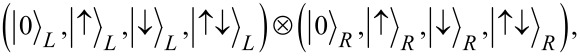


where the arrows denote the spin states of the electrons. We will use the notation 
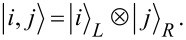
 We perform second-order Schrieffer–Wolff perturbation theory in the tunneling term *H*_T,SC_ to obtain the effective Hamiltonian for the 16-dimensional low-energy subspace. See Supporting Information File 1 for the derivation and the validity conditions. As a result of this procedure, we find that the QD-SC tunneling *H*_T,SC_ generates three coupling terms in the effective Hamiltonian: (i) a local (single-dot) pairing term, called local Andreev reflection (LAR), (ii) a non-local (interdot) pairing term, called crossed Andreev reflection (CAR), and (iii) an effective interdot tunneling term, called elastic co-tunneling (EC):

[9]
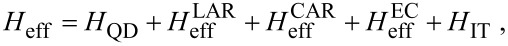


In *H*_eff_, the second and third terms read as

[10]
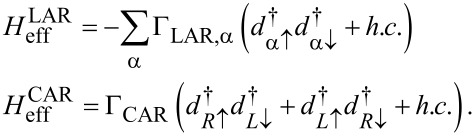


The effective parameters Γ_LAR,α_ and Γ_CAR_ are related to each other on the level of the presented model, i.e., neglecting the spatial separation of the QDs (see, e.g., [52] and Supporting Information File 1). However, for the remainder of this work, we will consider Γ_LAR,α_ and Γ_CAR_ as independent parameters, since the CAR mechanism is expected to be suppressed, when a finite distance between the QDs is introduced [74].

The EC term 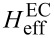 describes single-electron tunneling between the QDs via SC. This term, in contrast to the LAR and CAR coupling, has a strong dependence on the on-site energies ε*_L_*, ε*_R_* of the QDs. For example, the EC matrix element coupling the 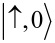 and 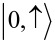 states is well approximated by

[11]
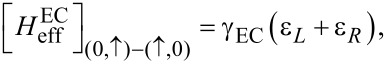


whereas the matrix element coupling the 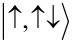 and 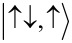 states is

[12]
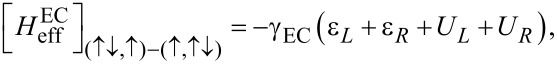


where the strength of the EC mechanism is characterized by the dimensionless parameter γ_EC_ = Γ_CAR_/Δ. See Supporting Information File 1 for the derivation and for the complete list of the matrix elements.

To illustrate the dependence of these matrix elements on the QD on-site energies, we plot the two matrix elements shown in Equation 11 and Equation 12 in Figure 1b, using Δ = 5*U* and Γ_CAR_ = 0.1*U*. The two matrix elements, determined from second-order perturbation theory, plotted in Figure 1b vanish at ε*_L_* = −ε*_R_* and ε*_L_* + *U_L_* = −ε*_R_*−*U_R_*, respectively. Of course, if such a contribution vanishes, then higher-order terms neglected here may actually be important.

Importantly, fermion parity and spin are conserved in our effective model. This implies that the 16-dimensional effective Hamiltonian has a block structure; more precisely, there are six orthogonal subspaces that are not mixed by the effective Hamiltonian. These invariant subspaces are shown in Figure 1c. The first invariant subspace (singlet - S, left panel of Figure 1c) contains the five spin-singlet states with even number of electrons on the QDs: the empty and the doubly occupied states 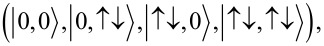 and the spin-singlet combination of the (1,1) states, 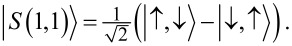 The second and third invariant subspaces (doublet - D, right panel of Figure 1c) contain the states with odd number of electrons. Since we do not account for a magnetic field, these eight states are decomposed into two invariant subspaces with different total spin *z* component


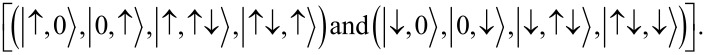


Each energy eigenvalue in one doublet subspace has an equal partner in the spectrum of the other doublet subspace. The three spin-triplet combinations of the (1,1) states, i.e., 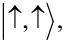 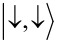 and


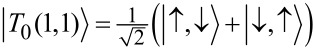


remain uncoupled from each other and from the other invariant subspaces, and these three states have the same energy eigenvalue.

In Figure 1c, the arrows visualize the tunneling-induced matrix elements coupling the basis states of the effective Hamiltonian. Note that in Figure 1c, we use the singlet–triplet basis instead of the product basis. The tunneling processes giving rise to the coupling matrix elements indicated by red arrows on Figure 1c, are illustrated in Figure 1d. For instance, one of the LAR matrix elements corresponds to transferring a Cooper pair from the SC to QD_R_ through a virtual intermediate state, in which one electron occupies QD_R_ and one quasiparticle is present in the SC. The analogous CAR matrix element corresponds, again, to extracting a Cooper pair from the SC, but in this case the electrons end up in different QDs. Both the EC and the IT matrix elements correspond to the transfer of an electron from one QD to the other. In the case of EC, there is an intermediate virtual state with one quasi-particle in the SC, but in the case of IT, the tunneling is direct. The difference between EC and IT processes results an important difference of their matrix elements: for EC, they depend on the on-site energies [see, e.g., Equation 11 and Equation 12], while for IT they do not (see Equation 8).

In what follows, we will rely on the numerically obtained eigenvalues *E*_χ_ and eigenstates 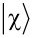 of the effective Hamiltonian *H*_eff_ of Equation 9. Furthermore, we will use *U* = *U_L_* = *U_R_* as the energy unit, Γ_LAR,_*_L_* = Γ_LAR,_*_R_* = 0.25*U*, and will focus on the parameter range Γ_CAR_ 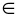 [0,0.1]*U*, γ_EC_ 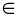 [0,0.15], *t*_LR_ 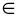 [0,0.1]*U*. To convert our results to physical units, we can use, e.g., *U* = 1 meV; then the above numbers correspond to an experimentally realistic parameter set.

## Ground-state Properties

**Figure 2:**
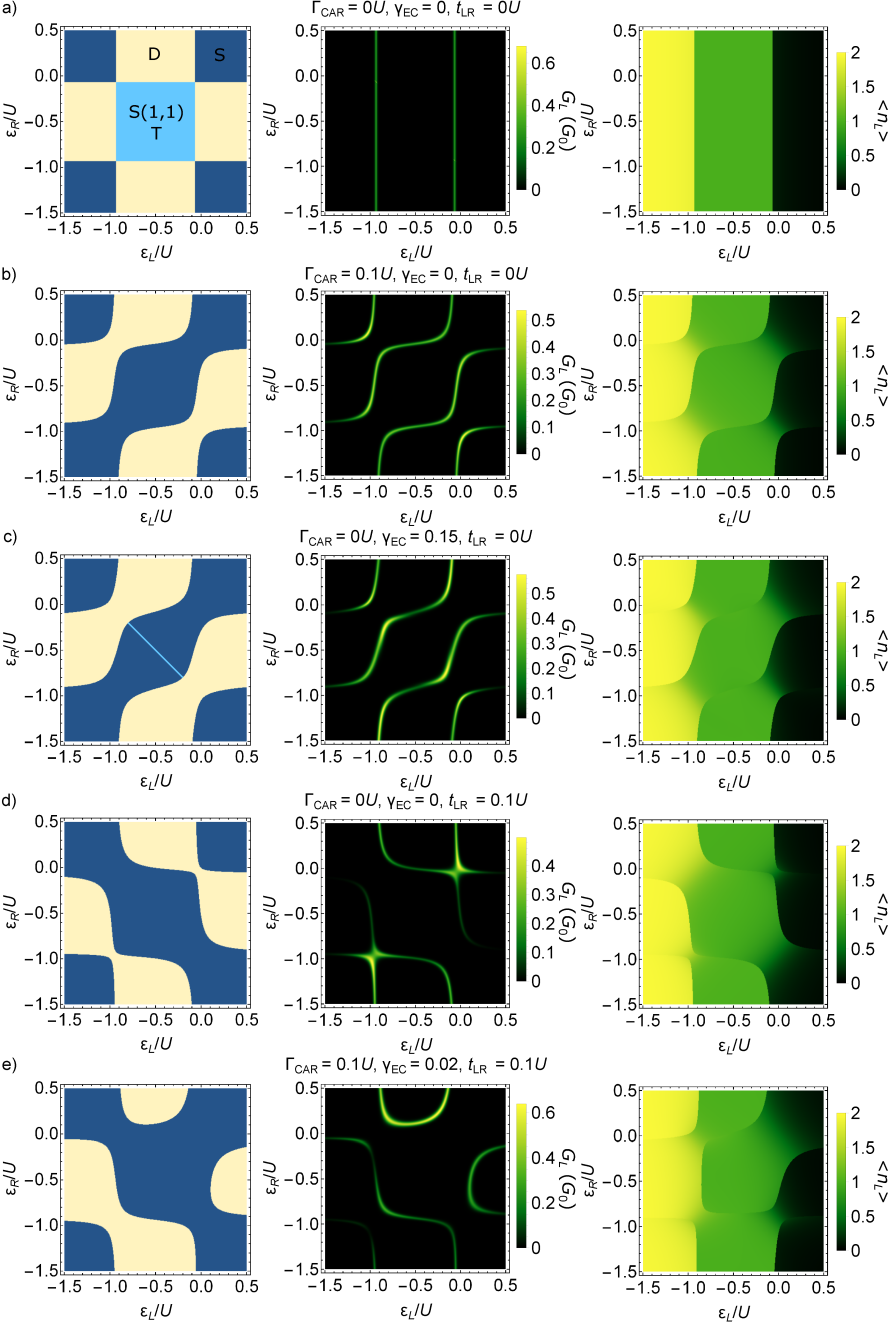
Phase diagram and related measurable quantities of the QD–SC–QD system. Phase diagram (left column), zero-bias conductance *G_L_* of the left lead N_L_ (middle column), and average electron occupation 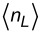 of QD_L_ (right column) are shown, for different non-local coupling configurations: a) without non-local couplings, b) only CAR, c) only EC, d) only IT, e) all three.

Here we analyze the ground-state properties of the effective Hamiltonian *H*_eff_, namely, the ground-state degeneracy, the fermion parity and the average electron occupations of the QDs, as functions of the on-site energies ε*_L_* and ε*_R_*, for different values of the non-local coupling terms CAR, EC and IT (see Figure 2a–e). First we analyze the fingerprints of the three non-local coupling mechanisms, one-by-one, and finally we show an example where all coupling terms are finite. We show that although CAR and EC couple different states (see Figure 1b), they produce rather similar phase diagrams, but IT can be clearly distinguished from the previous two. In an experimental situation when one of these mechanisms is dominant, our results can be used to identify that dominant mechanism. We also note that in an actual experiment, it is challenging to effectively tune the ratio of these parameters.

The left column of Figure 2 shows the phase diagram, the middle column shows the zero-bias conductance of QD_L_, and the right column shows the average electron occupation of QD_L_ for different Γ_CAR_, γ_EC_ and *t*_LR_ values. Note that for all cases the local pairing term is finite, Γ_LAR,_*_L_* = Γ_LAR,_*_R_* = 0.25*U*. In this subsection, we discuss the phase diagram and the average electron occupation, and return to the zero-bias conductance results later, after introducing the transport model.

The phase diagrams of the QD–SC–QD system display the dependence of two ground-state properties on the on-site energies ε*_L_* and ε*_R_*: (i) the degree of degeneracy of the ground state, and (ii) the fermion parity of the ground state. For example, Figure 2a shows the phase diagram without non-local couplings, i.e., Γ_CAR_ = γ_EC_ = *t*_LR_ = 0. Different colors correspond to different ground-state degeneracies: Yellow denotes a two-fold degenerate ground state, dark blue denotes a non-degenerate ground state, and light blue denotes a four-fold degenerate ground state. The ground state has even (odd) fermion parity in the dark blue and light blue (yellow) regions. Note that such a phase diagram should be regarded as the generalization of the stability diagrams of non-superconducting double QDs, see, e.g., Figure 2 of [75].

In Figure 2a, where non-local couplings are absent, the two QDs are independent, thus the phase boundaries are vertical and horizontal lines. (Recall that interdot Coulomb repulsion is neglected.) The dark blue regions correspond to a singlet (S), unique ground state, where both QDs are in the bonding combination of the states 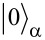 and 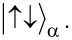 Note that in the absence of non-local couplings, the state 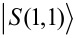 remains uncoupled from the other four states in the singlet subspace, and degenerate with the three triplets 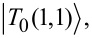 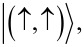 and 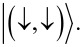 As a consequence, the ground-state degeneracy in the central, light blue region is four-fold. The two ground states of the yellow regions are drawn from the doublet (D) subspace of Figure 1b [35].

In general, if a non-local coupling is turned on, then the light blue regions (four-fold degenerate ground state) disappear, see Figure 2b–e. The reason for that is as follows. Due to the non-local coupling, the state 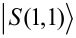 couples to the other four singlet states 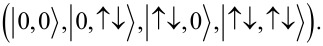 Therefore, the energy of the lowest-energy singlet eigenstate will be lower than the energy of the triplets. This mechanism leaves two possibilities for the ground state: non-degenerate singlet, or a two-fold degenerate doublet. The only exception is a line, when EC is the only finite non-local mechanism, where the four-fold degeneracy is preserved, which we discuss below.

The case of finite CAR coupling is presented in Figure 2b with Γ_CAR_ = 0.1*U*. Besides the disappearance of the four-fold degenerate light blue region, another difference compared to Figure 2a is the merging of the singlet-phase regions along the diagonal (i.e., the ε*_L_* = ε*_R_* line) and the merging of the doublet-phase regions parallel to the diagonal. These features are consequences of the CAR coupling, and can be understood via simple perturbative arguments.

For example, consider the top left quadruple point in the phase diagram of Figure 2a. At this point, the most relevant doublet states are 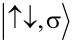 and 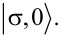 These states are coupled directly by CAR, as shown in Figure 1b; as a consequence, the bonding combination of these will form the doublet ground state, with an energy lowered by approximately Γ_CAR_ due to the non-local coupling. On the other hand, the most relevant singlet states are 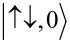 and *S*(1,1), which are not coupled directly by CAR, see Figure 1b. In conclusion, the doublet ground state will have a lower energy than the singlet ground state in the top left quadruple point, explaining the merge of the doublet phase parallel to the diagonal in Figure 2b. Similar considerations apply to the other three quadruple points of Figure 2a.

Consider now the case when the only non-local coupling mechanism in the setup is EC. For this case, we can infer the ground-state character from a perturbative consideration similar to the one above. This consideration yields the expectation that the singlet (doublet) phases would merge along (parallel to) the skew diagonal (i.e., the line ε*_R_* = *U* − ε*_L_*) of the phase diagram, in contrast to the case of CAR. However, the numerically evaluated phase diagram for this case (γ_EC_ = 0.15), plotted in Figure 2c, shows that the phase diagram is actually very similar to Figure 2b.

To understand this surprising feature, we have to (i) go beyond the previous first-order perturbative analysis, and (ii) go beyond the qualitative arguments based on the selection rules of Figure 1b, i.e., taking into account the on-site energy dependence of the EC coupling matrix elements, exemplified in Equation 11 and Equation 12. For example, consider the top right quadruple point in Figure 2a. Here, the lowest-energy states are dominantly 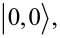 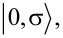 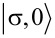 and 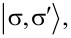 from which the doublet states 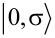 and 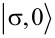 should be coupled directly by EC (see Figure 1b), but the corresponding matrix elements 
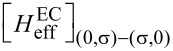
 are proportional to ε*_L_* + ε*_R_* (see Equation 11), and hence are strongly suppressed. On the other hand, the two other states, 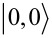 and 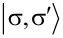 are coupled by LAR and EC in second order via intermediate states, by (cf. Figure 1b)


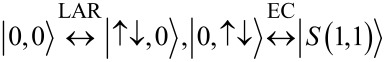


which includes the 
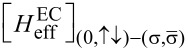
 matrix element, which is not suppressed. Hence, at the top right quadruple point, this second-order hybridization results in a lowered energy of the singlet ground state. Similar considerations explain the features of the phase diagram in Figure 2c at all four quadruple points.

A difference between Figure 2b and Figure 2c is the presence of a light blue skew diagonal line at the central region of Figure 2c. Along this line, the EC matrix element 
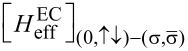
 vanishes (see Equation S13 in Supporting Information File 1), and therefore the state 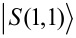 is decoupled from the other singlet states, and remains degenerate with the triplets, preserving the four-fold degeneracy.

Figure 2d shows the phase diagram for the case when the only non-local coupling mechanism is IT, for *t*_LR_ = 0.1*U*. Here, the singlet and doublet phases merge parallel to the skew diagonal. This is explained by arguments analogous to the first-order perturbative considerations outlined above, keeping in mind that the non-local coupling matrix elements of IT do not depend on the QD on-site energies.

As we discussed in the introduction the nature of the EC coupling is model dependent, in certain cases it has no dependence on the on-site energies, hence it is indistinguishable from IT coupling. In such cases the phase diagram is sufficient to distinguish between the CAR and the EC/IT couplings.

In conclusion, if we assume that only one non-local coupling mechanism is present, then CAR and on-site energy dependent EC produce rather similar phase diagrams, but they can be clearly distinguished from the case of IT.

All phase diagrams of Figure 2a–d are symmetric in two ways: (i) for the transformation (ε*_L_*,ε*_R_*) 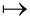 (ε*_R_*,ε*_L_*), and (ii) for the transformation (ε*_L_*,ε*_R_*) 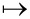 (−*U_L_*−ε*_L_*,−*U_R_*−ε*_R_*). The property (i), which we call the left–right symmetry, originates from the symmetric choice of local parameters, i.e., *U_L_* = *U_R_*, Γ_LAR,_*_L_* = Γ_LAR,_*_R_*. The property (ii) is the result of a particle–hole symmetry of the system, as discussed in the following.

A particle–hole transformation converts the filled electron states to empty ones and vice versa, i.e., exchange the role of creation and annihilation operators. Eight different particle–hole transformations are introduced and discussed in Supporting Information File 1. One example is the transformation (iv) in Table S1 of Supporting Information File 1, corresponding to the mapping 
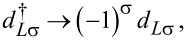
 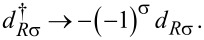 Each transformation can be represented as a unitary transformation *W* on the 16-dimensional Fock space. For each Hamiltonian terms *H*(ε*_L_*,ε*_R_*), these transformations connect the inverted points of the phase diagram (see Supporting Information File 1 for details), namely

[13]
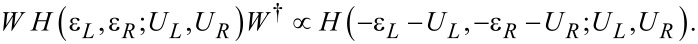


These transformations correspond to an inversion in the phase diagram to the central point (ε*_L_*,ε*_R_*) = (−*U_L_*/2,−*U_R_*/2), usually called particle–hole symmetric point. We say that the transformation *W* is the particle–hole symmetry of the Hamiltonian term *H*, if Equation 13 is an equality. All coupling Hamiltonians 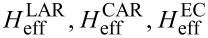 and *H*_IT_ have a few such particle–hole symmetries, but each term has a different set of those, see Table S1 of Supporting Information File 1. If there exists a single particle–hole transformation that is a particle–hole symmetry of all coupling terms forming the Hamiltonian, then the phase diagram (along with other quantities) reflects the particle–hole symmetry. For example, the phase diagram in Figure 2d, where IT is the only non-local coupling mechanism, shows particle–hole symmetry, since transformation (iv) in Table S1 of Supporting Information File 1 is a particle–hole symmetry of the Hamiltonian from which 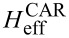 and 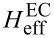 are omitted.

Finally, consider the general case, having all non-local couplings finite, Γ_CAR_ = *t*_LR_ = 0.1*U* and γ_EC_ = 0.02. Figure 2e shows the phase diagram for this case. First, the left–right symmetry is apparent that is still a consequence of the left–right symmetric choice of the parameter values. Second, the particle–hole symmetry is absent in Figure 2e. This is consistent with the fact that none of the eight particle–hole transformations is a particle–hole symmetry of all terms in this general Hamiltonian (see Table S1 in Supporting Information File 1).

The phase boundaries between ground states of different fermion parities can be mapped by low-energy transport [75]. The expected zero-bias conductance, with peaks following the even-odd phase boundaries, is illustrated by the middle column of Figure 2 and will be discussed in more detail below. A drawback of transport analysis is that the coupling to the electrodes lead to the broadening of conductance peaks (which is neglected from the model presented here). One may reduce broadening by decreasing the coupling at the price of decreasing the currents, too.

Charge sensing [76–77] is another method to map out the boundaries of the phase diagram, as we illustrate in the right column of Figure 2. A charge sensor is usually engineered to be mostly sensitive to the average electron occupation of one of the quantum dots, say, QD_L_. Compared to the conductance measurement through the QD–SC–QD system, charge sensing has the advantage of conceptual simplicity, and the measurability without additional N leads attached to the QD–SC–QD system; but might have the disadvantage of a more complex device design, since the charge sensor is an additional device element. Similar methods, yielding information related to average electron occupation, are based on reflectometry with electromagnetic radiofrequency signals [78–79] or microwave resonators [80–81]; those are not discussed further in this work.

The ground-state average electron occupation in QD_L_ is expressed as


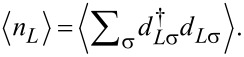


We plot 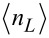 as the function of the on-site energies ε*_L_* and ε*_R_* in the right column of Figure 2, for the parameter values providing the previously discussed phase diagrams.

In the absence of non-local couplings (Figure 2a), the QDs are independent, therefore 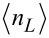 does not depend on ε*_R_*. We emphasize, since it is not apparent in the 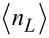 density plot in Figure 2a, that values of 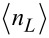 are not restricted to the integer values 0, 1 and 2. This is because the LAR mechanism provides coherent coupling within a given fermion-parity sector between states with different electron numbers, see Figure 1b. In fact, 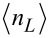 as a function of ε*_L_* slightly decreases in the yellow (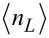 ≈ 2) and black (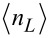 ≈ 0) regions in the right panel of Figure 2a, its value is strictly 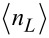 = 1 in the green region, and jumps abruptly at the boundaries.

The non-local couplings are switched on in Figure 2b–e. Similarly to Figure 2a, the average electron occupation 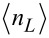 decreases as ε*_L_* is increased, and the jump locations in 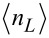 follow the phase boundaries. The jumps are more pronounced along the vertical phase boundaries, i.e., in a charge-sensing experiment the measurement of QD_L_ maps out the vertical phase boundary lines more efficiently. Due to the finite non-local couplings, the variation of the average electron occupation within a given fermion-parity sector is smooth, as shown in Figure 2b–e.

Due to the left–right symmetry, for all cases presented here, the average electron occupation of QD_R_, that is, 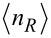, can be obtained by mirroring 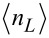 to the diagonal. Therefore, in the 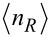 map the horizontal phase boundary lines are more pronounced. This allows for the measurement of the phase boundaries by measuring the occupation of the two QDs independently.

In this subsection, we have shown that on contrary to expectations, the CAR and the EC mechanisms produce rather similar phase diagrams as functions of ε*_L_* and ε*_R_*, but IT can be clearly distinguished from the previous two mechanisms. Furthermore, the measurement of the average electron occupation of the QDs allows for determining the phase boundaries, even in the absence of normal electrodes tunnel-coupled to the QD–SC–QD system.

## Transport Calculation

As pointed out earlier, a charge-sensing measurement is demanding, since the addition of the charge sensor complicates device fabrication. However, the ground-state phase diagrams discussed above can also be explored experimentally by electronic transport measurements, utilizing two additional N leads besides the SC lead (see Figure 1a) and low bias voltages. We will demonstrate this using the results shown in the middle column of Figure 2. Transport measurements using a sufficiently large bias voltage allow to determine energy gaps above the ground state. We will show (see below Figure 4) that such finite-bias measurements can distinguish the CAR-dominated and EC-dominated cases, i.e., the two cases that are not distinguished by the ground-state properties shown in Figure 2b,c.

The transport setup we wish to describe is shown in Figure 1a. Throughout this work, we assume that the SC lead is grounded, μ_SC_ = 0, and the two N leads are biased symmetrically, μ*_NL_* = μ*_NR_* = μ*_N_*, with the convention that for positive (negative) μ*_N_*, the electrons tend to flow into (out from) the SC lead.

Our transport model is based on the effective Hamiltonian *H*_eff_ of Equation 9 describing the QD–SC–QD system. In addition, here we also take into account the N-lead Hamiltonians and the lead-QD tunneling Hamiltonians, that is, *H*_N_ + *H*_T,N_. We describe the electronic transport in this device using a classical master equation, where the tunnel rates between the N leads and the QD–SC–QD system are obtained perturbatively, from Fermi’s golden rule.

The classical master equation describes the time evolution of the occupation probabilities *P*_χ_(*t*) of the 16 energy eigenstates 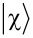 of *H*_eff_, and reads

[14]
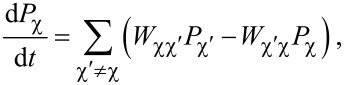


with the normalization condition 
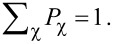
 The transition rates


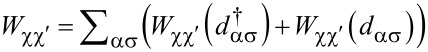


are obtained from the leading-order term in Fermi’s golden rule as

[15] $$\begin{array}{l} W_{\chi \chi^{\prime }}\left(d_{\alpha \sigma }^{†}\right)=\Gamma_{N\alpha }{\left|\langle \chi |d_{\alpha \sigma }^{†}|\chi^{\prime }\rangle \right|}^{2}f\left(E_{\chi }-E_{\chi^{\prime }}-\mu_{N\alpha }\right),\\ W_{\chi \chi^{\prime }}\left(d_{\alpha \sigma }\right)=\Gamma_{N\alpha }{\left|\langle \chi |d_{\alpha \sigma }|\chi^{\prime }\rangle \right|}^{2}f\left(E_{\chi }-E_{\chi^{\prime }}+\mu_{N\alpha }\right), \end{array}$$

where α 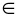 {*L*,*R*} is the lead index, 
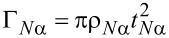
 is a characteristic tunneling rate between the normal lead N_α_ and the SC–QD–SC system, ρ*_Nα_* is the density of states at the Fermi energy in the lead N_α_, and 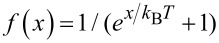 is the Fermi function.

We calculate the stationary (d*P*_χ_/d*t* = 0) solution of Equation 14 to obtain the steady-state occupation probabilities *P*_χ_, and use the latter to evaluate the steady-state current in lead *N*_α_ via

[16]
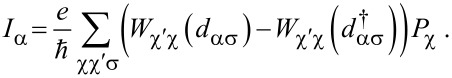


Since we use a leading-order Fermi’s golden rule, each non-zero transition rate corresponds to the tunneling of a single electron between a lead and the QD–SC–QD system, therefore connects states with different fermion parities. The differential conductance of lead N_α_ is calculated by numerically differentiating the current by the chemical potential of the normal leads, i.e.,


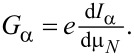


We plot the conductance in the units of the conductance quantum *G*_0_ = 2*e*^2^/*h*.

In the following, we will use Γ*_NL_* = Γ*_NR_* = Γ*_N_* = 0.005*U* and *k*_B_*T* = 0.005*U*. If our energy unit is *U* = 1 meV, then Γ*_N_* = 5 μeV. Note that in our model, the currents and differential conductances are simple linear functions of Γ*_N_*, since we employ a leading-order Fermi’s golden rule. Furthermore, the broadening of the conductance resonances in our results is caused only by the finite temperature of *k*_B_*T* = 0.005*U* ≈ *k*_B_·60 mK, since the presented model neglects the life-time broadening. Experimentally, life-time broadening of the conductance resonances might be dominant over the thermal broadening. In this case, the line width can be reduced by decreasing the tunnel coupling to the N leads, at the price of suppressing the currents.

## Results of the Transport Simulation

Here, we present the results we obtained from the transport model of the previous section. First, we discuss how to experimentally map the phase boundaries using zero-bias conductance measurements, as presented in Figure 2. Second, we demonstrate that finite-bias differential conductance measurements provide a means to distinguish a system dominated by CAR from one dominated by EC. Third, we demonstrate and analyze the appearance of negative differential conductance in our setup, which is often attributed to the triplet blockade [52–53] and the CAR mechanism; here we show that not only CAR but any of the three non-local coupling mechanism can result in triplet blockade and a corresponding negative differential conductance.

### Zero-bias conductance

The zero-bias conductance of lead N_L_, *G_L_* is shown in the middle column of Figure 2, for the five previously discussed cases, i.e., in the absence of non-local couplings Figure 2a, when having only one of them turned on (Figure 2b–d), and the general case of all three having finite values (Figure 2e). For all five cases, *G_L_* shows a resonant enhancement along the phase boundaries. The conductance in Figure 2b–e shows further enhancement in the vicinity of the quadruple points seen in the left panel of Figure 2a.

Due to the left–right symmetric choice of the parameter values, similarly to the average electron occupation, the conductance *G_R_* of QD_R_ can be obtained from *G_L_* by mirroring the latter to the diagonal ε*_L_* = ε*_R_* line.

In conclusion, the zero-bias measurement is a sufficient tool to locate the phase boundaries in the ε*_L_*–ε*_R_* plane, and to determine whether the interaction between the dots is due to the proximity of the superconductor (CAR or EC dominates over IT), or not (IT dominated).

### Finite-bias conductance

Here, first we show how the presence non-local couplings affect the finite-bias transport for a general case, then we show that CAR and EC provide different fingerprints, and therefore these measurements can distinguish between a CAR-dominated setup and an EC-dominated setup.

**Figure 3:**
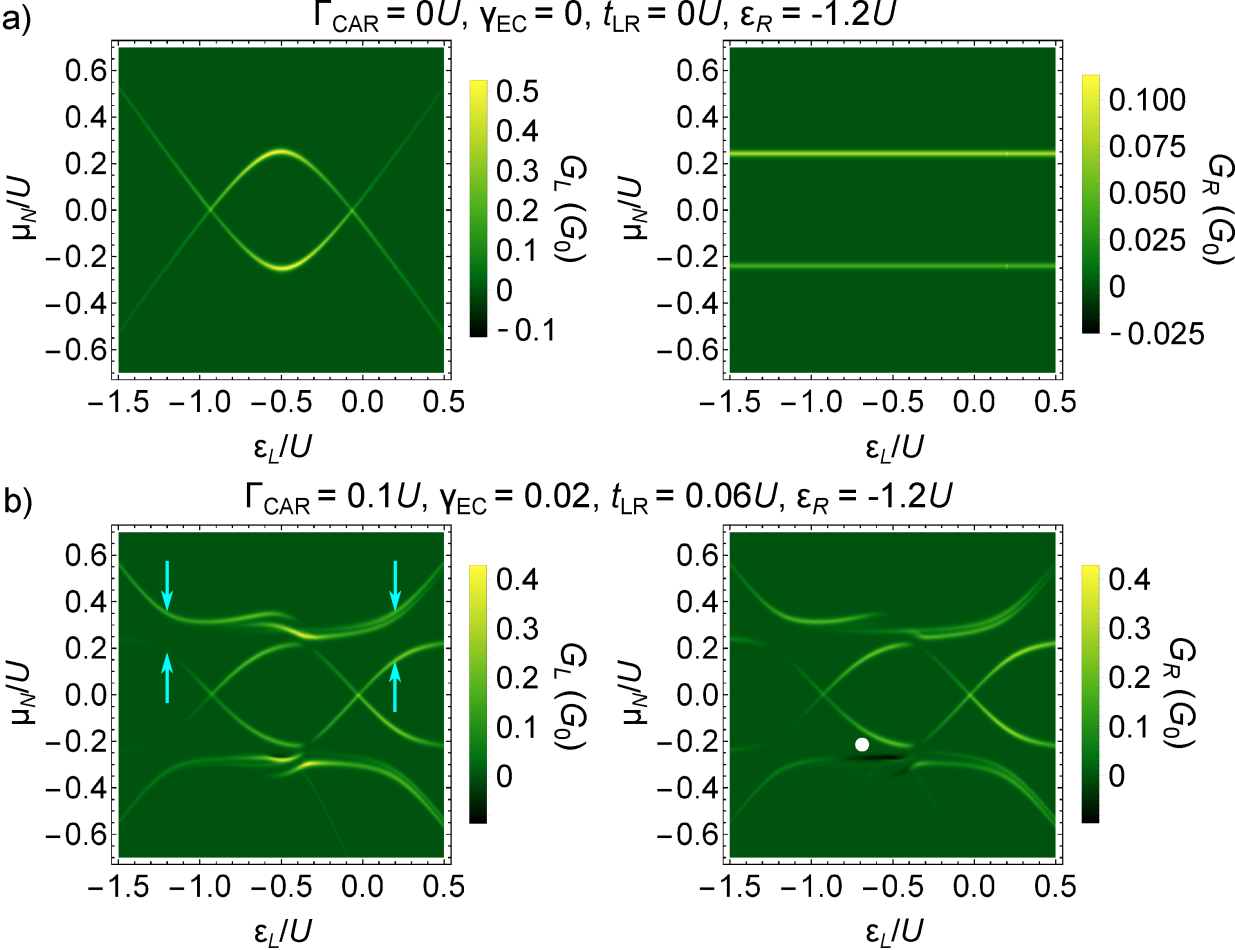
Hybridization of the two quantum dots of the Cooper-pair splitter due to the non-local coupling mechanisms. Differential conductances *G_L_* and *G_R_* of the Cooper-pair splitter are shown in the left and right panels, respectively, for *U_L_* = *U_R_* = *U*, Γ_LAR_*_,L_* = Γ_LAR_*_,R_* = 0.25*U*, and fixed ε*_R_* = −1.2*U*, as a function of ε*_L_* and bias voltage μ*_N_*. a) Without non-local couplings. Differential conductance shows the single-dot Andreev bound states formed on the dots. b) With non-local couplings. Their presence leads to the hybridization of the Andreev bound states on the dots, indicated by the appearance of anticrossings marked with arrows. Parameters: Γ_CAR_ = 0.1*U*, γ_EC_ = 0.02, *t*_LR_ = 0.1*U*. For better visibility, different color scales were used for different panels.

Figure 3a shows the finite-bias conductances *G_L_* and *G_R_* of the two leads, in the absence of non-local couplings, for a fixed value ε*_R_* = −1.2*U*, as the function of ε*_L_* and the bias voltage μ*_N_*. Figure 3b shows the same quantities, for a general case when all three non-local couplings are switched on.

Without non-local couplings (Figure 3a), Andreev bound states (ABSs) are formed on each QD due to the coupling to the superconductor. The ABSs on the two QDs are independent. In Figure 3a, the conductance *G_L_* in the left panel shows an eye-shaped resonance with two crossing points at zero bias. This characteristic resonance is the usual fingerprint of an ABS in a single QD (see, e.g., [35]). The resonance maps the excitation energy between the two lowest-energy eigenstates of the QD_L_-SC subsystem: the bonding linear combination of the singlet-like state, 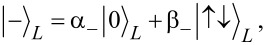 and the doublets, 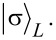 In the central region, the doublets are the ground states, while on the sides, the ground state is the singlet 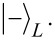 Note that the higher-energy antibonding singlet state, 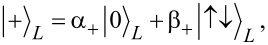 can be accessed from the doublet states, but the corresponding conductance resonance lies out of the energy window used here. Note also that in our model, transitions between the two singlet states are forbidden, since these two states both have even fermion parity.

As noted earlier, the positive μ*_N_* sides of the finite-bias conductance plots of Figure 3 correspond to transport by adding electrons from the N_L_ to QD_L_, while the negative μ*_N_* sides represent the opposite processes. Although the finite-bias conductance plots are not symmetric to the zero-bias axis, the conductance lines are positioned symmetrically, since (i) the spectrum does not depend on μ*_N_* and (ii) transition between the eigenstates with different fermion parity is possible either by adding or by removing one electron. Generally, the tunnel rate for adding an electron is different from the tunnel rate for removing an electron, therefore the heights of the conductance resonances at positive and negative bias are different.

A complete transport cycle constitutes of the transport of two electrons, e.g., for positive bias, the first electron enters the QD with positive energy, bringing it to an excited state, while the second electron enters with negative energy, making the system relax back to its ground state. These two electrons, which reside in the leads at the beginning of the cycle, are absorbed by the SC as a Cooper pair by the end of the cycle. Analogously, for negative bias, the first (second) electron leaves the QD with negative (positive) energy.

The finite-bias conductance plots in the absence of non-local couplings (Figure 3a) exhibit an inversion symmetry, *G_L_*(ε*_L_*, μ*_N_*) = *G_L_*(−*U_L_* − ε*_L_*, −μ*_N_*). We attribute this to the particle–hole symmetry discussed above, with an extension of the particle–hole transformations to the lead electrons. In the presence of non-local couplings, if the Hamiltonian is particle–hole symmetric, then the inversion symmetry *G_L_*(ε*_L_*, ε*_R_*, μ*_N_*) = *G_L_*(−*U_L_* − ε*_L_*, −*U_R_* − ε*_R_*, −μ*_N_*) would be reflected in the finite-bias conductance plots (not shown).

In the finite-bias conductance plot of QD_R_ only two horizontal lines are present (see the right panel in Figure 3a). This is due to the independence of the QDs: ε*_L_* has no effect on the transport via QD_R_. The position and the amplitude of the lines is the same as the ones in the left panel of Figure 3a, at ε*_L_* = −1.2*U*.

When the non-local couplings are switched on, they induce a crosstalk between the two QDs, resulting in a strong modification of the finite-bias conductance plots. This is shown in Figure 3b, for a general case with all three non-local coupling being finite, Γ_CAR_ = 0.1*U*, γ_EC_ = 0.02 and *t*_LR_ = 0.06*U*. The previously discussed features, i.e., the eye-shaped resonances in *G_L_* and the horizontal lines in *G_R_*, appear in both finite-bias conductance plots of Figure 3b. In addition, anticrossings between conductance resonances are openened by the non-local couplings wherever the excitation energies of the two uncoupled dots would coincide. The two most pronounced anticrossings are marked with arrows in the left panel of Figure 3b, open at ε*_L_* = ε*_R_* = −1.2*U* and ε*_L_* = −*U* − ε*_R_* = 0.2*U*.

**Figure 4:**
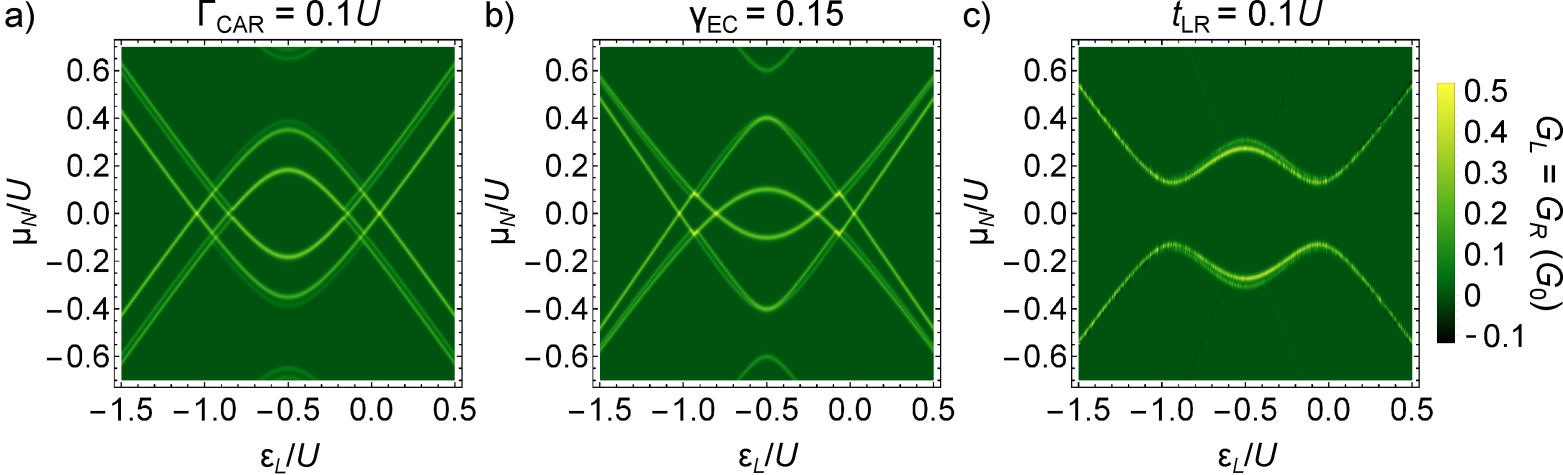
Differential conductance as a fingerprint of the non-local coupling mechanism. Differential conductance of the QD–SC–QD system is shown, for the previously used parameters *U_L_* = *U_R_* = *U* and Γ_LAR_*_,L_* = Γ_LAR_*_,R_* = 0.25*U*, along the skew-diagonal line ε*_R_* = −*U* −ε*_L_*. Only one non-local coupling mechanism is non-zero for each panel. a) Γ_CAR_ = 0.1*U*, b) γ_EC_ = 0.15, c) *t*_LR_ = 0.1*U*. The dependence of the splitting on ε*_L_* is qualitatively different in a) and b), implying that experimental conductance data can be used to determine whether CAR or EC is the dominant non-local coupling mechanism.

The analysis of the conductance resonances as functions of the on-site energies allows one to distinguish between the coupling terms. This is shown in Figure 4, where the finite-bias conductance along the skew diagonal ε*_R_* = −*U* − ε*_L_* of the phase diagram is shown, for only one finite non-local coupling. Note that along this skew diagonal, we have *G_L_* = *G_R_*. Figure 4a, Figure 4b and Figure 4c) show the case of CAR, EC and IT coupling, with Γ_CAR_ = 0.1*U*, γ_EC_ = 0.15 and *t*_LR_ = 0.1*U*, respectively. The conductance resonances in Figure 4a and Figure 4b show an appreciable splitting, which is absent in the right panel. The size of the splitting is constant for CAR, while for EC it is the largest at the particle–hole symmetric point, ε*_L_* = ε*_R_* = −*U*/2 and decreases further from it. This difference between the finite-bias conductance can used to identify whether the dominant non-local coupling mechanism is CAR or EC. As expected from the qualitatively different nature of the phase diagrams of Figure 2b and Figure 2c compared to Figure 2d, the case of IT is also qualitatively different in terms of finite-bias conductance (Figure 4a and Figure 4b compared to Figure 4c).

Further finite bias data and discussion on the features of Figure 3 and Figure 4 is provided in Supporting Information File 1.

### Triplet blockade

**Figure 5:**
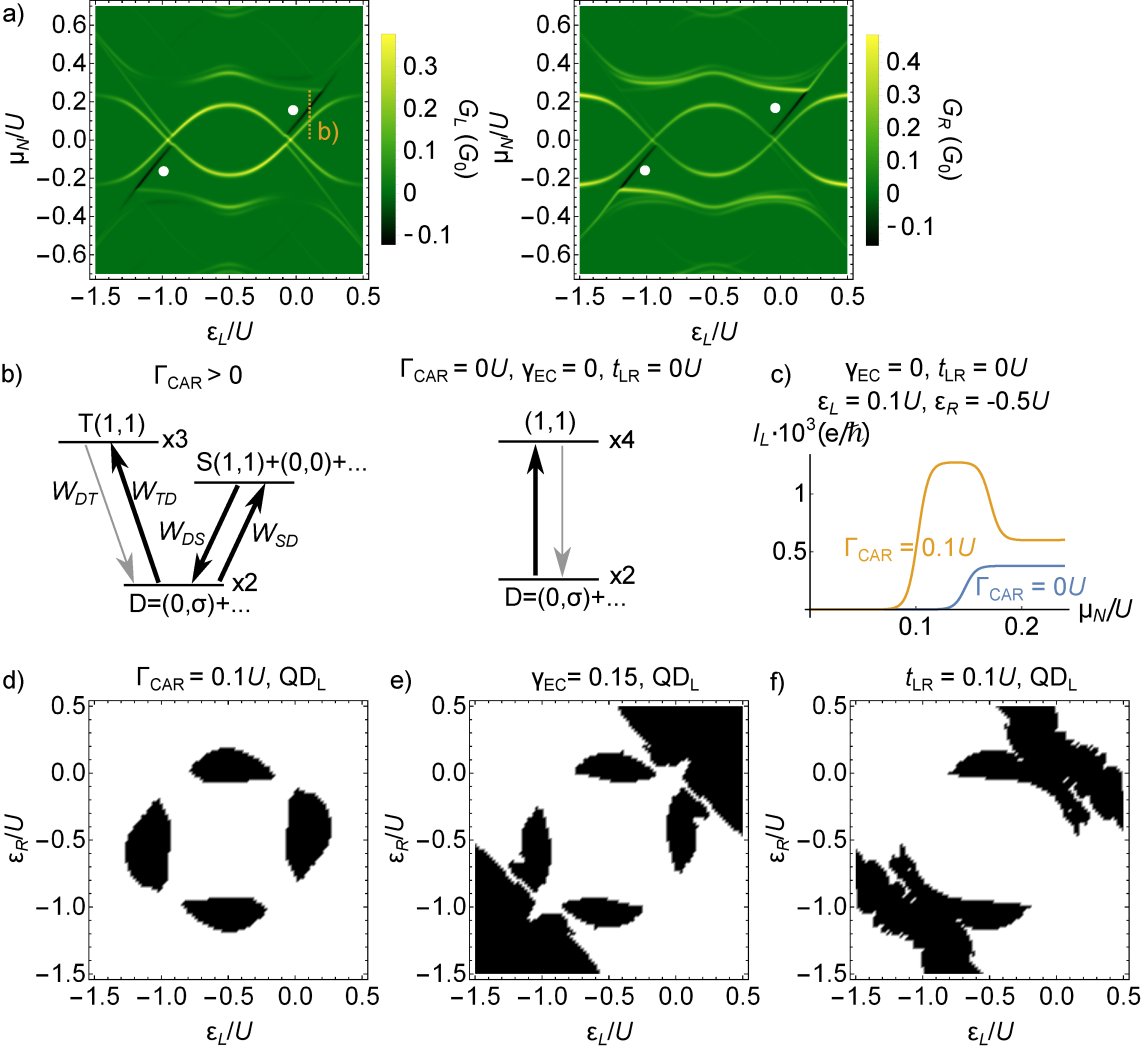
Triplet blockade and negative differential conductance in the Cooper-pair splitter. a) Finite-bias differential conductances *G_L_* and *G_R_* for Γ_CAR_ = 0.1*U*, γ_EC_ = 0, *t*_LR_ = 0, at ε*_R_* = −*U*/2. b) Levels and transitions taking part in triplet blockade. c) Current–voltage dependence in lead *N_L_* with (orange) and without (blue) CAR coupling, along the dotted vertical line in panel a). Introducing a non-local coupling mixes the states, hence a well conducting channel is opened. d–f) On-site-energy regions where triplet-blockade-induced negative differential conductance is presented in black: d) Γ_CAR_ = 0.1*U*, e) γ_EC_ = 0.15, f) *t*_LR_ = 0.1*U*.

In certain cases, the finite-bias conductance plots show line-shaped regions of negative differential conductance (NDC). Examples, marked with white circles, are shown in the right panel of Figure 3b and in Figure 5. Further examples are shown on Figure S1 and S2 in Supporting Information File 1.

The NDC lines appear when a so-called blocking state becomes energetically available as μ*_N_* reaches its excitation energy. The blocking state starts to be populated, but the rates of transitions out of this state are small, therefore the population is accumulating in the blocking state, reducing the current. When the blocking states are spin-triplets, then this effect is called triplet blockade. The triplet blockade is often referred to as a hallmark of the CAR coupling in the literature. It is argued that the spin incompatibility of the triplet state in the QDs and the spin-singlet Cooper pairs in the SC prohibit CAR coupling of the QDs [52–53]. Here, we show that the presence of NDC lines is not exclusive for the CAR, but they can appear for all three non-local couplings.

Figure 5a shows an example of triplet-blockade-related NDC lines in the CAR coupled case, for Γ_CAR_ = 0.1*U* and ε*_R_* = −*U*/2. Let us focus on the cut at ε*_L_* = 0.1*U*, marked with the dashed orange vertical line. For these parameter values, we show the relevant energy levels in the left panel of Figure 5b. The ground state is a doublet,


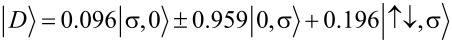
 
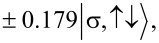


and the first excited state is a singlet, 
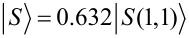
 − 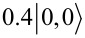 − 
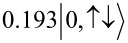
 − 
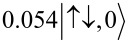
 + 
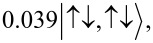
 with a dominant 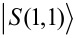 contribution. The second excited level is the threefold degenerate triplet level. All excited states are reachable from the ground state via electron tunneling, but tunneling transitions between two different excited states are forbidden, as both states have even fermion parity.

In the left panel of Figure 5b, we illustrate the tunneling transitions (arrows) relevant for electron transport at this parameter point. If the bias voltage is sufficiently large to induce a transition to the singlet excited state, then a well-conducting transport channel opens up, characterized by the uphill rate *W_SD_* and the downhill rate *W_DS_*. In this transport cycle, the uphill transition, 
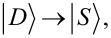
 is dominated by the 
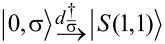
 process, while the downhill transition, 
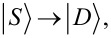
 is dominated by 
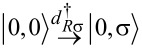
 and 
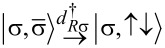
 processes. *W_SD_* = 0.383Γ*_N_* and *W_DS_* = 0.297Γ*_N_* are the relevant transition rates.

When the bias voltage is further increased, then the triplet states are also populated, see Figure 5b. In this case, the uphill transition from the ground state to the triplet is dominated by the 
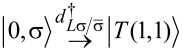
 process, with the rate *W_TD_* = 0.465Γ*_N_* rate. The downhill transitions from the triplets are possible, and their rates are dominated by the 
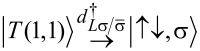
 and 
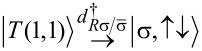
 processes, but these rates are small, *W_DT_* = 0.019Γ*_N_*, more than one order of magnitude smaller than the other transition rates. This small downhill transition rate results in the accumulation of the population in the triplet states, and the reduction the singlet and doublet populations that would provide efficient conduction. Therefore, the net effect of the triplet states becoming available upon increasing the bias voltage is the reduction of the current, and as a consequence, the appearance of the NDC lines in the differential conductivity.

We show the level diagram and the transitions, in the absence of CAR coupling, in the right panel of Figure 5b. In this case, due to the absence of CAR, the 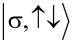 component is missing from the ground states, and the 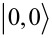 is missing from the excited states, which are parts of the two main downhill processes enabling the 
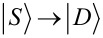
 transition, as we have seen above. Therefore all four, now degenerate, (1,1) states block the transport, as illustrated on the right panel of Figure 5b.

The comparison of the two cases is shown in Figure 5c, where the current *I_L_* in the left lead is plotted as the function the bias voltage μ*_N_* with blue (orange) line without non-local coupling (with Γ_CAR_ = 0.1*U*) for the same ε*_L_* = 0.1*U* and ε*_R_* = −*U*/2 as above. In the presence of finite CAR, as the bias voltage is increased from zero, first a well conducting channel opens around μ*_N_* ≈ 0.1*U*, and the current jumps down at μ*_N_* ≈ 0.17*U* due to the triplet blockade. In contrast, in the absence of non-local couplings, above μ*_N_* ≈ 0.15*U* the current is comparably small as in the blockaded case. Note that in the presence of CAR coupling, the triplet excitation is shifted to somewhat higher energy, since the energy of the ground state is lowered due to the non-local coupling.

The orange line in Figure 5c indicates a negative differential conductance. However, the current does not drop to zero but forms a finite plateau for μ*_N_* *>* 0.17*U*, i.e., the triplet blockade is ‘incomplete’, and there is a finite ‘leakage current’. As discussed by Trocha and Weymann in [53], this leakage current is due to the fact that double occupancy of the QDs is allowed in this model. In contrast, in models neglecting double occupancy, e.g., by assuming infinite on-site Coulomb repulsion *U*→∞, this leakage current vanishes exactly [52].

As we have seen, the presence of the high current due to the well-conducting singlet–doublet channel requires that (i) the singlet energy is brought below the energy of the triplets, and (ii) the 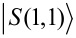 state is mixed with the empty or double occupied states. These conditions can be induced not only by CAR, but by any of the three non-local couplings described here. Thus, for all the three cases, triplet-blockade-induced NDC lines can appear. In Figure 5c–e we map the on-site energy regions where the triplet blockade is present for the three different couplings. We marked with black those (ε*_L_*,ε*_R_*) values where there exists a bias voltage, at which the absolute value of the current *I_L_* decreases, and the triplet occupation of the QD–SC–QD system increases by at least 0.25 simultaneously. The black regions of Figure 5d–f are not symmetric for mirroring to the diagonal ε*_L_* = ε*_R_* line, thus the NDC lines are present in different (ε*_L_*,ε*_R_*) regions for QD_L_ and QD_R_. Note that due to the left-right symmetry the NDC maps calculated from *I_R_* would be the same as the presented ones mirrored to the diagonal.

In our setup, NDC can also appear in cases where it is not caused by triplet blockade. In the main text, we have been focusing on triplet-blockade-induced NDC, but an example for non-triplet-induced NDC is show in Supporting Information File 1 in Figure S1b.

In conclusion, we have shown that the triplet blockade is not exclusive for CAR, but can appear also in the presence of any of the three non-local coupling mechanisms. In fact, a blockade can arise even in the absence of non-local couplings, even though the singlet state is also blocking in that case. The presence of a non-local coupling makes the singlet state well conducting, and makes the triplet blockade effect easily observable in a transport experiment by the appearance of NDC lines.

## Conclusion

We have analyzed the spectrum of a QD–SC–QD system in the presence of different non-local coupling mechanisms: CAR, EC and IT. Our aim was to calculate the effects of these on measurable quantities (phase diagrams, average electron occupations, zero-bias and finite-bias conductance), with the goal of identifying features that are characteristic for each non-local coupling mechanism.

The phase diagram of the system can be mapped via charge-sensing or zero-bias conductance measurements. We find that CAR and EC produces very similar phase diagrams, thus measuring the phase diagram alone would not allow one to distinguish between these two non-local coupling mechanisms. However, IT produces a qualitatively different phase diagram, and hence it should be straightforward to identify if the non-local couplings are dominated by IT in a device. Furthermore, we have demonstrated that finite-bias measurements could be used to distinguish between the cases when CAR or EC dominates the non-local couplings.

In the literature, triplet blockade is often linked to the presence of the CAR mechanism. Here we have shown that the suppression of the current due to the population of the triplet states is not specific to CAR coupling. In fact, such a current suppression can appear even without any non-local mechanism present in the device. In the presence of non-local processes, the current suppression can be interpreted as a triplet blockade, and it is observed via NDC lines, and this effect is not unique for CAR, but EC and IT can also generate it.

We expect that the results presented here will facilitate the accurate characterization of hybrid superconductor–quantum dot devices, which are likely to be used as building blocks of future conventional and topological quantum-information schemes.

## Supporting Information

File 1 Derivation of the effective Hamiltonian, details of the particle-hole symmetry and additional transport data.
