# Supplementary material for: Transport signatures of an Andreev molecule in a quantum dot–superconductor–quantum dot setup
Source: Beilstein J Nanotechnol. 2019 Feb 6;10:363–78. doi: 10.3762/bjnano.10.36 (PMC6369982; doi:10.3762/bjnano.10.36)
Supplement: File 1 — Derivation of the effective Hamiltonian, details of the particle-hole symmetry and additional transport data. [file Beilstein_J_Nanotechnol-10-363-s001.pdf]

BEILSTEIN JOURNAL OF NANOTECHNOLOGY

Supporting Information

for

Transport signatures of an Andreev molecule in a quantum dot–superconductor–quantum dot setup

Zoltán Scherübl, András Pályi and Szabolcs Csonka

Beilstein J. Nanotechnol. **2019,** *10,* 363–378. doi:10.3762/bjnano.10.36

Derivation of the effective Hamiltonian, details of the particle-hole symmetry and additional transport data

License and Terms: This is a supporting information file under the terms of the Creative Commons Attribution License (http://creativecommons.org/licenses/by/4.0). Please note that the reuse, redistribution and reproduction in particular requires that the authors and source are credited.
The license is subject to the *Beilstein Journal of Nanotechnology* terms and conditions: (https://www.beilstein-journals.org/bjnano)

Derivation of the effective Hamiltonian

In the main text, we used a low-energy effective Hamiltonian to describe the quantum dot – superconductor – quantum dot (QD-SC-QD) setup, being valid in the limit of large SC gap Δ, when the quasiparticle states of the SC can only be filled virtually. The effective Hamiltonian can be derived using second order quasi-degenerate perturbation theory (see e.g. Appendix B of Ref. [1]), by integrating out the quasiparticle states. The unperturbed Hamiltonian is $H_{\mathrm{QD}} + H_{\mathrm{SC}}$, the perturbation is $H_{\mathrm{T,SC}}$, and the effective Hamiltonian is expressed via

$$\left[H_{\mathrm{eff}}^{(2)}\right]_{i,j} = \frac{1}{2}\sum_{l}\left[H_{T,SC}\right]_{il}\left[H_{T,SC}\right]_{lj}\left(\frac{1}{E_i^{(0)} - E_l^{(0)}} + \frac{1}{E_j^{(0)} - E_l^{(0)}}\right). \tag{S1}$$

Here, the summation index l runs over unperturbed energy eigenstates containing one quasiparticle, the i and j indices run over the 16 low-energy quasiparticle-free unperturbed energy eigenstates, and $E_i^{(0)}$ are the corresponding energy eigenvalues. To ensure the validity of the perturbative treatment, the relevant, quasiparticle-free, states must have lower energy than the virtual ones with a single quasi particle. For the on-site energy window which we study in this work, namely, $-1.5U < \varepsilon_L$, $\varepsilon_R < 0.5U$, this requirement translates to $\Delta > 3.5U$.

Tunneling gives rise to three terms in H_{eff}. The first term is called *local Andreev reflection* (LAR). It represents processes where the electron number in one of the QDs is changed by 2 (e.g. hybridizes $|0,0\rangle$ and $|0,\uparrow\downarrow\rangle$), because two electrons forming a Cooper pair in the SC are transferred to the same QD. The second term is called *crossed Andreev reflection* (CAR). It represents processes where the number of electrons on both QDs change by 1 (e.g., $|0,0\rangle \leftrightarrow |\uparrow,\downarrow\rangle$), because a Cooper pair is split between the two QDs. The third term is called *elastic cotunneling* (EC). It represents processes where the total number of electrons on the QDs remains the same, but one electron tunnels between the QDs via the superconductor (e.g., $|0,\uparrow\rangle \leftrightarrow |\uparrow,0\rangle$).

Note that the EC processes couple the same pairs of states as the interdot tunneling (IT), still EC has different effects on the energy eigenstates and transport properties, because of the pronounced dependence of its matrix elements on the on-site energies, as it will be discussed below.

LAR matrix elements

Here, we derive an exemplary matrix element for LAR, coupling the $|0,0\rangle$ and $|\uparrow\downarrow,0\rangle$ states, in detail. The first tunneling event splits a Cooper pair in SC, one of the electron tunnels to $\mathrm{QD_L}$, while the other electron will stay in the superconductor as a quasiparticle. The second tunneling event brings the system back to the low-energy subspace by annihilating the quasiparticle and creating a second electron on $\mathrm{QD_L}$. The corresponding matrix element is

$$[H_{\mathrm{eff}}]_{(0,0),(\uparrow\downarrow,0)} = \frac{1}{2} t_{SL}^2 \sum_{k\sigma} u_k v_k \left(\frac{1}{0-\varepsilon_L - E_k} + \frac{1}{2\varepsilon_L + U_L - \varepsilon_L - E_k} \right), \tag{S2}$$

where $E^{(0)}_{(0,0)} = 0$, $E^{(0)}_{(\uparrow\downarrow,0)} = 2\varepsilon_L + U_L$, and the energy of the intermediate virtual state is $\varepsilon_L + E_k$. The sum for the momentum k can be written as an energy integral,

$$[H_{\mathrm{eff}}]_{(0,0),(\uparrow\downarrow,0)} = \frac{1}{2} t_{SL}^2 \rho_0 \int_{-D}^{D} d\varepsilon_S \frac{\Delta}{\sqrt{\Delta^2+\varepsilon_S^2}} \left(\frac{1}{\varepsilon_L + U_L - \sqrt{\Delta^2+\varepsilon_S^2}} - \frac{1}{\varepsilon_L + \sqrt{\Delta^2+\varepsilon_S^2}} \right), \tag{S3}$$

where ρ_0 is the normal density of states of the SC, assumed to be constant and D is the bandwidth of the SC, and the definitions of the Bogoliubov-amplitudes (see Eq. 4 in the main text) were used. Generally, this integral cannot be performed analytically. However, in the large Δ limit, the typical ε_L and U_L values are small compared to the quasiparticle energy, therefore they can be neglected in the denominators. Then, the integration can be carried out analytically, resulting in

$$[H_{\mathrm{eff}}]_{(0,0),(\uparrow\downarrow,0)} = -t_{SL}^2 \rho_0 \int_{-D}^{D} d\varepsilon_S \frac{\Delta}{\Delta^2+\varepsilon_S^2} = -2t_{SL}^2 \rho_0 \arctan\left(\frac{D}{\Delta}\right) \overset{D\to\infty}{\to} -\pi t_{SL}^2 \rho_0 =: \Gamma_{LAR,L}. \tag{S4}$$

With the above approximation, all other matrix elements of LAR have the same form, i.e. $\Gamma_{LAR,\alpha} =$

$-\pi t_{S\alpha}^2\rho_0$, which can be summarized as

$$H_{\text{eff}}^{\text{LAR}} = -\sum_{\alpha} \Gamma_{LAR,\alpha} \left(d_{\alpha\uparrow}^{\dagger} d_{\alpha\downarrow}^{\dagger} + h.c. \right). \tag{S5}$$

Note: To check the validity of the approximation applied between Eqs. (S3) and (S4), we have numerically integrated Eq. (S3), and found that the difference between the numerical and approximate values of the matrix element is less than 10% for $\Delta > 5U_L$, and the variation of the matrix element is less than 3% in the $-1.5U < \varepsilon_L < 0.5U$ window (not shown here).

CAR matrix elements

The CAR mechanism is similar to the LAR, with a difference that the second electron is transferred to the opposite QD as the first one. Therefore, the matrix elements have a similar form, the difference is that both tunneling amplitudes appear in the result. Following a derivation similar to the previous one, we find that the CAR effective Hamiltonian can be written as

$$H_{\text{eff}}^{\text{CAR}} = \Gamma_{\text{CAR}} \left(d_{R\uparrow}^{\dagger} d_{L\downarrow}^{\dagger} + d_{L\uparrow}^{\dagger} d_{R\downarrow}^{\dagger} + h.c. \right), \tag{S6}$$

where $\Gamma_{\text{CAR}} = -\pi t_{SL} t_{SR} \rho_0$.

Within the framework our model, where the spatial separation of the QDs is neglected Γ_{CAR} is linked to the strength of the LAR couplings, i.e. $\Gamma_{\text{CAR}} = \sqrt{\Gamma_{\text{LAR,L}} \cdot \Gamma_{\text{LAR,R}}}$. However taking into account the finite separation of the QDs Γ_{CAR} become suppressed by the distance (see e.g. [2]), hence we treat them as an independent parameters.

EC matrix elements

The EC mechanism transfers one electron from one of the QDs to the other through a quasiparticle state in the SC. Here we show the derivation of the $|0,\uparrow\rangle \leftrightarrow |\uparrow,0\rangle$ matrix element, noting that further terms can be obtained similarly. There are two different sequences of underlying processes that build up the EC mechanism. In the first sequence, the electron tunnels from QD_R to the SC, where

it occupies a quasiparticle state, and then tunnels out to $\mathrm{QD_L}$. In the second sequence, a Cooper pair is split, the up-spin part tunneling out to $\mathrm{QD_L}$, while the down-spin remains in the SC as a quasiparticle, and then in the second tunneling event the quasiparticle in annihilated by the electron from $\mathrm{QD_R}$ as they together form a Cooper-pair in the SC. Having two processes leads to the doubling of terms in the matrix element:

$$\left[H_{\mathrm{eff}}^{\mathrm{EC}}\right]_{(0,\uparrow)-(\uparrow,0)} = \frac{1}{2} t_{SL} t_{SR} \sum_k \left(\frac{u_k^2}{\varepsilon_L - E_k} + \frac{u_k^2}{\varepsilon_R - E_k} + \frac{v_k^2}{\varepsilon_L + E_k} + \frac{v_k^2}{\varepsilon_R + E_k} \right) \tag{S7}$$

Using the same approximations as previously, i.e. neglecting $\varepsilon_{L/R}$ next to E_k, the matrix element vanishes:

$$\left[H_{\mathrm{eff}}^{\mathrm{EC}}\right]_{(0,\uparrow)-(\uparrow,0)} = -t_{SL} t_{SR} \sum_k \frac{u_k^2 - v_k^2}{E_k} = -t_{SL} t_{SR} \rho_0 \int_{-D}^{D} d\varepsilon_S \frac{\varepsilon_S}{\Delta^2 + \varepsilon_S^2} \equiv 0 \tag{S8}$$

As this approximation led to a vanishing result, we do the calculation without the approximation. In fact, the sum in Eq. (S7) can be converted into an integral which can be performed analytically, resulting in

$$\left[H_{\mathrm{eff}}^{\mathrm{EC}}\right]_{(0,\uparrow)-(\uparrow,0)} = 2 t_{SL} t_{SR} \rho_0 \left[\arctan\left(\frac{D}{\sqrt{\Delta^2 - \varepsilon_L^2}} \right) \frac{\varepsilon_L}{\sqrt{\Delta^2 - \varepsilon_L^2}} + \arctan\left(\frac{D}{\sqrt{\Delta^2 - \varepsilon_R^2}} \right) \frac{\varepsilon_R}{\sqrt{\Delta^2 - \varepsilon_R^2}} \right]. \tag{S9}$$

Taking the infinite-bandwidth limit, $D \to \infty$, this result simplifies to

$$\left[H_{\mathrm{eff}}^{\mathrm{EC}}\right]_{(0,\uparrow)-(\uparrow,0)} = \pi t_{SL} t_{SR} \rho_0 \left(\frac{\varepsilon_L}{\sqrt{\Delta^2 - \varepsilon_L^2}} + \frac{\varepsilon_R}{\sqrt{\Delta^2 - \varepsilon_R^2}} \right). \tag{S10}$$

One can further simplify the formula by neglecting ε_α next to Δ in the denominators, yielding

$$\left[H_{\mathrm{eff}}^{\mathrm{EC}}\right]_{(0,\uparrow)-(\uparrow,0)} \approx \pi t_{SL} t_{SR} \rho_0 \frac{\varepsilon_L + \varepsilon_R}{\Delta} = \Gamma_{CAR} \frac{\varepsilon_L + \varepsilon_R}{\Delta}. \tag{S11}$$

The main difference compared to the LAR, CAR and IT mechanisms is the dependence of the matrix element on the on-site energies. On the $\varepsilon_L = -\varepsilon_R$ line, this EC matrix element vanishes; it is possible that methods using less approximations (e.g., higher-order perturbation theory) yield non-vanishing contributions.

Eq. S11 implies that the strenghts of CAR and EC mechanisms are not independent. We introduce the dimensionless quantity

$$\gamma_{\mathrm{EC}} = \Gamma_{\mathrm{CAR}}/\Delta, \tag{S12}$$

to characterize the strength of the EC mechanism. Even though in our model, and most probably also in an actual experiment, the strengths of CAR and EC are linked, in the main text we treat Γ_{CAR} and γ_{EC} as independent parameters to analyze their effect separately.

With derivations similar to the previous one, the list of the non-zero matrix elements of the EC term of the effective Hamiltonian are as follows:

$$\begin{aligned}
\left[H_{\mathrm{eff}}^{\mathrm{EC}}\right]_{(0,\uparrow)-(\uparrow,0)} &= \gamma_{EC}\left(\varepsilon_L+\varepsilon_R\right) \\
\left[H_{\mathrm{eff}}^{\mathrm{EC}}\right]_{(0,\downarrow)-(\downarrow,0)} &= \gamma_{EC}\left(\varepsilon_L+\varepsilon_R\right) \\
\left[H_{\mathrm{eff}}^{\mathrm{EC}}\right]_{(\uparrow,\downarrow)-(0,\uparrow\downarrow)} &= \gamma_{EC}\left(\varepsilon_L+\varepsilon_R+U_R\right) \\
\left[H_{\mathrm{eff}}^{\mathrm{EC}}\right]_{(\downarrow,\uparrow)-(0,\uparrow\downarrow)} &= -\gamma_{EC}\left(\varepsilon_L+\varepsilon_R+U_R\right) \\
\left[H_{\mathrm{eff}}^{\mathrm{EC}}\right]_{(0,\uparrow\downarrow)-(\uparrow,\downarrow)} &= \gamma_{EC}\left(\varepsilon_L+\varepsilon_R+U_L\right) \\
\left[H_{\mathrm{eff}}^{\mathrm{EC}}\right]_{(0,\uparrow\downarrow)-(\downarrow,\uparrow)} &= -\gamma_{EC}\left(\varepsilon_L+\varepsilon_R+U_L\right) \\
\left[H_{\mathrm{eff}}^{\mathrm{EC}}\right]_{(\uparrow,\uparrow\downarrow)-(\uparrow\downarrow,\uparrow)} &= -\gamma_{EC}\left(\varepsilon_L+\varepsilon_R+U_L+U_R\right) \\
\left[H_{\mathrm{eff}}^{\mathrm{EC}}\right]_{(\downarrow,\uparrow\downarrow)-(\uparrow\downarrow,\downarrow)} &= -\gamma_{EC}\left(\varepsilon_L+\varepsilon_R+U_L+U_R\right).
\end{aligned} \tag{S13}$$

Particle-hole transformations and particle-hole symmetry

In the main text, we discussed that the different terms of the Hamiltonian might have particle-hole symmetry (PHS), which can be generated by various particle-hole transformations (PHTs). Here we will show how the individual terms of the Hamiltonian transform under different PHTs.

To analyze the presence and consequences of the PHS of our effective Hamiltonian, it is instructive to rewrite the on-site terms using the normal ordered particle number operators:

$$\tilde{H}_{\mathrm{QD}} = H_0 + H_{\mathrm{INT}} = \sum_{\alpha=L,R} \sum_{\sigma} \tilde{\varepsilon}_\alpha : n_{\alpha\sigma} : + \sum_{\alpha=L,R} U_\alpha : n_{\alpha\uparrow} :: n_{\alpha\downarrow} :, \tag{S14}$$

where $: n_{\alpha\sigma} := d^\dagger_{\alpha\sigma} d_{\alpha\sigma} - \frac{1}{2}$ is the normal ordered particle particle number operator. $\tilde{H}_{\mathrm{QD}}$ differs in two ways from the original H_{QD}. First, the reference of the on-site energy is shifted to 'the center of the odd charge state', or 'particle-hole symmetric point', i.e., $\tilde{\varepsilon}_\alpha = \varepsilon_\alpha - U_\alpha/2$. Second, a trivial constant shift of energy levels is introduced. The other terms of the effective Hamiltonian remain unchanged.

We consider here 8 different PHTs, detailed in the left part of Table. 1. In each transformation, the creation (annihilation) operators are replaced by annihilation (creation) operators. The difference of the transformations are the relative signs for different spins, different dots and whether the spin is flipped. Right part of Table. 1 shows how the different terms of Hamiltonians transform under the PHTs.

Table S1: Particle-hole transformations, and how the terms of the effective Hamiltonian of the Cooper pair splitter transform under the each transformation.

	$d^\dagger_{L\uparrow}$	$d^\dagger_{L\downarrow}$	$d^\dagger_{R\uparrow}$	$d^\dagger_{R\downarrow}$	H_0	H_{INT}	$H^{\mathrm{LAR}}_{\mathrm{eff}}$	$H^{\mathrm{CAR}}_{\mathrm{eff}}$	$H^{\mathrm{EC}}_{\mathrm{eff}}$	H_{IT}
i	$d_{L\uparrow}$	$d_{L\downarrow}$	$d_{R\uparrow}$	$d_{R\downarrow}$	-	+	-	-	+	-
ii	$d_{L\uparrow}$	$-d_{L\downarrow}$	$d_{R\uparrow}$	$-d_{R\downarrow}$	-	+	+	+	+	-
iii	$d_{L\uparrow}$	$d_{L\downarrow}$	$-d_{R\uparrow}$	$-d_{R\downarrow}$	-	+	-	+	-	+
iv	$d_{L\uparrow}$	$-d_{L\downarrow}$	$-d_{R\uparrow}$	$d_{R\downarrow}$	-	+	+	-	-	+
v	$d_{L\downarrow}$	$d_{L\uparrow}$	$d_{R\downarrow}$	$d_{R\uparrow}$	-	+	+	+	+	-
vi	$d_{L\downarrow}$	$-d_{L\uparrow}$	$d_{R\downarrow}$	$-d_{R\uparrow}$	-	+	-	-	+	-
vii	$d_{L\downarrow}$	$d_{L\uparrow}$	$-d_{R\downarrow}$	$-d_{R\uparrow}$	-	+	+	-	-	+
viii	$d_{L\downarrow}$	$-d_{L\uparrow}$	$-d_{R\downarrow}$	$d_{R\uparrow}$	-	+	-	+	-	+

All PHTs listed in Table. 1 preserves H_{INT}, but flips the sign of H_0. Therefore all PHTs introduced here connects the properties at $(\tilde{\varepsilon}_L, \tilde{\varepsilon}_R)$ to $(-\tilde{\varepsilon}_L, -\tilde{\varepsilon}_R)$, i.e. corresponding to an inversion with respect to $(\tilde{\varepsilon}_L = 0, \tilde{\varepsilon}_R = 0)$. Hence the $(\tilde{\varepsilon}_L = 0, \tilde{\varepsilon}_R = 0)$ point is called the particle-hole symmetric point.
The other coupling terms, $H_{\text{eff}}^{\text{LAR}}$, $H_{\text{eff}}^{\text{CAR}}$, $H_{\text{eff}}^{\text{EC}}$ and H_{IT} transform differently under different PHTs. We call a Hamiltonian particle-hole symmetric if there exists a PHT which preserves all terms of the Hamiltonian (except H_0).
A few examples: (1) If the complete Hamiltonian is the sum of the first five terms, i.e., IT is absent, then PHTs (ii) and (v) are PHSs, hence we expect to see the consequence of PHS on the measurable quantities, e.g., an inversion symmetry of the phase diagram (not shown). (2) If EC and IT are present simultaneously, then there is no PHT in Table 1. that would be a PHS. This is consistent with the fact that the phase diagram in Fig. 2e in the main text does not show inversion symmetry. (3) If LAR and IT are present in the Hamiltonian, then PHT (iv) is a PHS, ensuring the inversion symmetry of the phase diagram, as confirmed by Fig. 2d of the main text.
Our study focuses on a two-site model. One work in which the role of PHTs was elaborated on is Ref. [3], where Sznajd and Becker showed that the generalization of PHT (iii) in Table 1 is a PHS of the usual non-superconducting Hubbard-model on a bipartite lattice.

Additional data on finite bias transport simulation

In the main text we discussed that in general the non-local coupling terms hybridize the Andreev states on the separate QDs, leading to the appearance of anticrossings in the transport spectrum. Here we will show the similarities and the differences of the anticrossings originating form different coupling terms. Generally two dominating anticrossings open, one on diagonal, i.e. on the $\varepsilon_R = \varepsilon_L$ line and one on the skew-diagonal, $\varepsilon_R = -U - \varepsilon_L$, their relative size and their evolution as ε_α is tuned differ for each coupling mechanisms.
On Fig. S1 we show the differential conductance of the two side, G_L and G_R, in the absence of non-local couplings on panel (a) (same as Fig. 3a in the main text) and with only one coupling being finite on panels (b-d) ($\Gamma_{\text{CAR}} = 0.1U$ on panel (b), $\gamma_{\text{EC}} = 0.15$ on panel (b), $t_{\text{LR}} = 0.1U$ on panel (d)) as the

function of ε_L and μ_N for fixed $\varepsilon_R = -1.2U$. Fig. S2 shows the conductance for the three latter case along the diagonal and the skew-diagonal, to illustrate the evolution of the size of the anticrossings. In case of CAR coupling ($\Gamma_{\rm CAR} = 0.1U$) the more pronounced anticrossing is located on the skew-diagonal at $\varepsilon_L = 0.2U$, constituting of 3 conductance lines, appearing along the dashed line on Fig. S1b. Another smaller anticrossing is present on the diagonal ($\varepsilon_R = \varepsilon_L$) at $\varepsilon_L = -1.2U$, marked with gray dotted line.

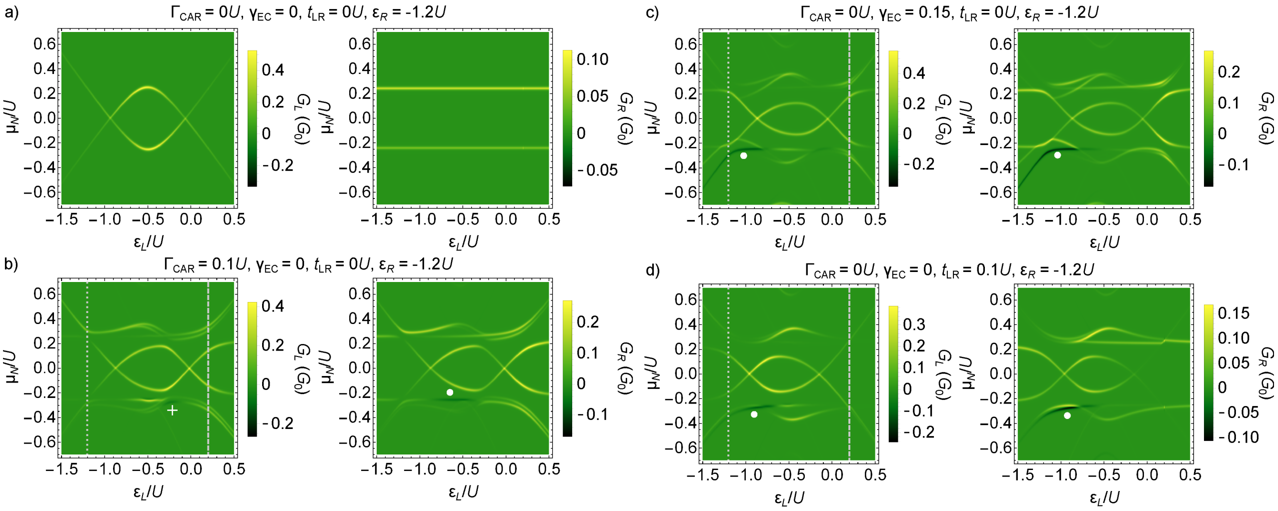

Figure S1: Differential conductance of the QD-SC-QD system to the N leads as a function of ε_L and μ_N for fixed $\varepsilon_R = -1.2U$, with $\Gamma_{LAR,L} = \Gamma_{LAR,R} = 0.25U$ a) without non-local couplings the usual single-QD ABS is formed on the dots, same as Fig. 3a in the main text., b) with $\Gamma_{\rm CAR} = 0.1U$, c) with $\gamma_{\rm EC} = 0.15$, with $t_{\rm LR} = 0.1U$. The presence of the non-local couplings lead to the hybridization of the ABSs on the QDs, indicated by the appearance of anticrossings. The gray dashed and dotted lines mark the correspondence with Fig. S2. The white circles mark the triplet blockade related NDC lines and the white + sign on panel b) marks a not triplet related NDC.

When ε_R is tuned, the size of the anticrossing positioned on the skew-diagonal remains the same, while for the one moving on the diagonal is maximal in the particle-hole symmetric point ($\varepsilon_L = \varepsilon_R = -U/2$) and decrease as the on-site energies are tuned away from this point. These two different evolution are shown on panel d) and a) of Fig. S2, respectively. The gray dashed and dotted lines also mark the correspondence of Fig. S1 and Fig. S2. Note that due to the symmetries for the special cuts, presented on Fig. S2 the conductance of the two sides are equal, $G_L = G_R$.

Explanation of the anticrossings: At $\varepsilon_L = 0.2U$, $\varepsilon_R = -1.2U$ the ground state of the QD-SC-QD system is a Signlet, dominantly $|gs\rangle \propto |0,\uparrow\downarrow\rangle + \ldots$ and the first two excited states are Doublets,

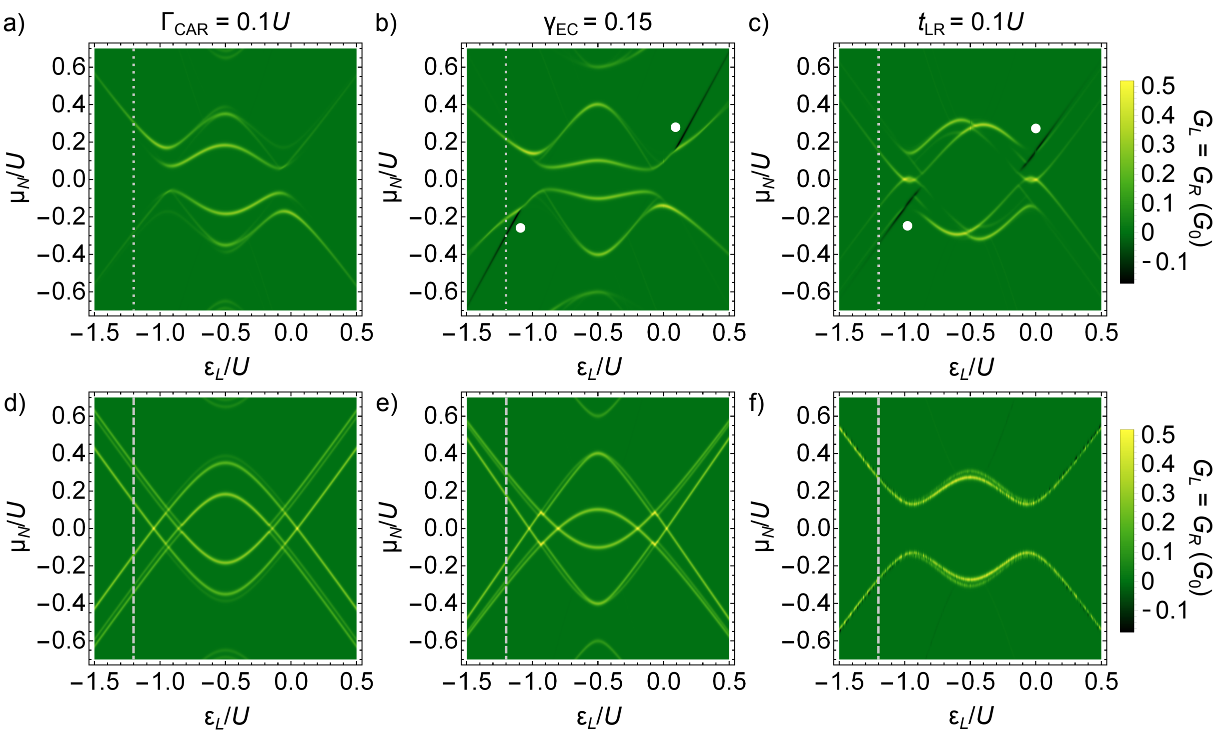

Figure S2: Differential conductance of the QD-SC-QD system along the diagonal, $\varepsilon_R = \varepsilon_L$ (panel a),b) and c)) and the skew-diagonal $\varepsilon_R = -U - \varepsilon_L$ lines (panel d) e) and f)) for different non-local parameters: a),d) with $\Gamma_{\text{CAR}} = 0.1U$, b),e) with $\gamma_{\text{EC}} = 0.15$ and c),f) with $t_{\text{LR}} = 0.1U$. $\Gamma_{LAR,L} = \Gamma_{LAR,R} = 0.25U$ as previously. The gray dashed and dotted lines mark the correspondence with Fig. S1. The size of anticrossing a), decrease b) increase, c),d) stays constant and e) decrease as ε_R is tuned away from the particle-hole symmetric point. f) the first two excitation are degenerate, there is no anticrossing present. Panel d-f) are the same as Fig. 4 in the main text.

$|es_{1/2}\rangle \propto |0,\sigma\rangle \pm |\sigma,\uparrow\downarrow\rangle + ...$, which are the bonding and antibonding combination of $|0,\sigma\rangle$ and $|\sigma,\uparrow\downarrow\rangle$ states, hybridized by the CAR. The lower and upper excitation lines of the three in the anticrossing corresponds to the $|gs\rangle \to |es_1\rangle$ and $|gs\rangle \to |es_2\rangle$ transitions.

The third, middle line running parallel with the upper one corresponds to the excitation to the third, singlet excited state, from the first one, $|es_1\rangle \to |es_3\rangle = |S(1,1)\rangle + ...$ The excitation energy for the transition is lower than the $|gs\rangle \to |es_2\rangle$ transition's. Note that this third excited state, $|es_3\rangle$ cannot be accessed from the ground state since it has the same parity.

At $\varepsilon_L = \varepsilon_R = -1.2U$ the ground state is dominantly $|gs\rangle = |\uparrow\downarrow,\uparrow\downarrow\rangle + ...$ and the excited states are $|es_1\rangle = |\sigma,\uparrow\downarrow\rangle + ...$ and $|es_2\rangle = |\uparrow\downarrow,\sigma\rangle + ...$ These two components cannot directly by coupled by CAR, since they have the same number of electrons. However they can be coupled in second order, using the fact that LAR mix these state with $|\sigma,0\rangle$ and $|0,\sigma\rangle$ respectively. The CAR matrix element

is finite between the $|\sigma,0\rangle$ ($|0,\sigma\rangle$) and $|\uparrow\downarrow,\sigma\rangle$ ($|\sigma,\uparrow\downarrow\rangle$) states. The absence of the direct coupling between the excited states results in the suppression of the size of the anticrossing compared to the one on the skew-diagonal.

The splitting of further conductance lines is comparable to the line width, which is further broadened in an experiment due to the coupling to the normal leads – which is neglected in the present model –, therefore we neglect the detailed analysis of the fine structure here.

In case of EC the anticrossings are positioned similarly as for CAR (see Fig. S1c with $\gamma_{\mathrm{EC}} = 0.15$), a larger one constituting of 3 conductance line positioned on the skew-diagonal (dashed line) and a smaller one on the diagonal (dotted line). The main difference between EC and CAR lies in behavior of the size of the anticrossing as ε_R is tuned. While the pronounced one have the same size for CAR (Fig. S2d), for EC is maximal in the particle-hole symmetric point and it decreases (see Fig. S2e) as ε_R is tuned away from this point. For the anticrossing positioned on the diagonal the behavior is strictly different from the case of CAR: On one hand the higher energy anticrossing line has a negative differential conductance (see the black lines on Fig. S2b) for $\varepsilon_L > 0$ (negative bias) and $\varepsilon_L < -U$ (positive bias). Furthermore the two excitation lines move apart as ε_L increased from 0 (or decreased below $-U$) for EC, while the distance between the two lines decreases for CAR (Fig. S2a). Note that second excitation is not visible for opposite bias directions as discussed previously on Fig. S2b.

The third conductance line in the anticrossing positioned on the skew-diagonal is originating from the excitation to the third excited state similarly to the case of CAR, but positioned at higher energy than the first two excitations for EC.

Interestingly for the anticrossing positioned on the diagonal the splitting disappears close to $\varepsilon_L = \varepsilon_R \approx -U$ (see Fig. S2b). At these parameters the hybridized excited states are dominantly formed from by the $|\sigma,\uparrow\downarrow\rangle$ and $|\uparrow\downarrow,\sigma\rangle$ states, which are coupled by the $\left[H_{\mathrm{eff}}^{\mathrm{EC}}\right]_{(\sigma,\uparrow\downarrow)-(\uparrow\downarrow,\sigma)}$ matrix element, which vanishes at $\varepsilon_L = \varepsilon_R = -U$ (see Eq. 12 of Supplementary Information File 1.).

Note that due to the different definition of Γ_{CAR} and γ_{EC} one cannot directly compare the magnitude of the splittings (see Eq. 11 Supplementary Information File 1.).

Finally Fig. S1d shows the effect of the IT, with $t_{\mathrm{LR}} = 0.1U$. On contrary to the CAR and EC, in this case only one anticrossing can be found – along the diagonal (dotted line) – and there is no anticrossing on the skew-diagonal (dashed line). The size of the anticrossing does not depend on the value of ε_L, except the $\varepsilon_L \approx -U/2$ region (see Fig. S2c). On the skew diagonal the first two excitations are exactly degenerate, independently of ε_R (see Fig. S2f).

As we have shown above the main, common signature of the non-local couplings is the appearance of anticrossings in the excitation spectrum of the QD-SC-QD system, and the main difference of the three couplings lie in the behavior of these anticrossings. For CAR and EC they are positioned similarly, but their size change differently when ε_R is changed, while for IT only one anticrossing is present. This difference in the anticrossings allows one to determine the dominant coupling term in an experiment.

A not triplet related NDC line is shown on Fig. S1b at $\varepsilon_L \approx -0.2U$, $\mu_N \approx -0.3U$ (marked with a white + sign), where the blocking occurs when the $|es_1\rangle \rightarrow |es_4\rangle$ transition becomes available, where $|es_1\rangle$ is a singlet, and $|es_4\rangle$ is a doublet state.

References

1. Winkler, R. *Spin-Orbit Coupling in Two-Dimensional Electron and Hole Systems*; Springer: Berlin Heidelberg, 2003.

2. Recher, P.; Sukhorukov, E. V.; Loss, D. *Phys. Rev. B* **2001**, *63*, 165314. doi:10.1103/PhysRevB.63.165314.

3. Sznajd, J.; Becker, K. W. *J. Phys.: Condens. Matter* **2005**, *17*, 7359. doi:10.1088/0953-8984/17/46/020.
